# Challenges in assessing national radiotherapy costs: application of the ESTRO-HERO model in Spain

**DOI:** 10.3389/fpubh.2024.1474376

**Published:** 2024-12-19

**Authors:** Julieta Corral, Manel Algara, Carles Muñoz-Montplet, Arantxa Eraso, Jordi Giralt, Noémie Defourny, Yolande Lievens, Josep Maria Borras

**Affiliations:** ^1^Catalonian Cancer Plan, Department of Health, Government of Catalonia, Barcelona, Spain; ^2^Bellvitge Biomedical Research Institute (IDIBELL), Hospitalet Barcelona, Barcelona, Spain; ^3^Radiation Oncology Service, Hospital del Mar Barcelona, Barcelona, Spain; ^4^Departament of Medicine and Life Science, Pompeu Fabra University, Barcelona, Spain; ^5^Department of Medical Physics and Radiation Protection, Catalan Institute of Oncology, Girona, Spain; ^6^Department of Medical Sciences, University of Girona, Girona, Spain; ^7^Radiation Oncology Service, Institut Català d’Oncologia - Girona, Girona, Spain; ^8^Radiation Oncology Service, Vall d'Hebron Barcelona Hospital Campus, Barcelona, Spain; ^9^Belgian Cancer Centre, Scientific Institute of Public Health (Sciensano), Brussels, Belgium; ^10^Department of Radiation Oncology, Ghent University Hospital, Ghent University, Ghent, Belgium; ^11^Faculty of Medicine and Health Sciences, Ghent University, Ghent, Belgium; ^12^Department of Clinical Sciences, University of Barcelona, Barcelona, Spain

**Keywords:** external beam radiotherapy, economic evaluation, costing model, time-driven activity based costing (TD-ABC), value-based healthcare

## Abstract

**Background and purpose:**

The aim was to estimate the cost of the external beam radiotherapy (EBRT) in public health care centers in Catalonia (Spain), according to the ESTRO-HERO costing model for 2018.

**Materials and methods:**

Personnel, equipment, and activity data from 2018 from the 11 RT centers were used, incorporating European mean values adapted to the Catalan context. Secondly, EBRT costs were estimated, incorporating 2023 fractionation technique and scheme usage percentages. Finally, complementary estimates were included: complementary planning examinations, stereotactic body radiation therapy (SBRT) fiducial markers, and hospital overhead costs.

**Results:**

In 2018, EBRT cost was estimated at EUR 42.2 M for all patients in the region. Directly related treatment activities represented 69.0% of the total cost, while support and non-directly related EBRT activities accounted for 20.2 and 10.8%, respectively. Mean radical treatment cost varied from €1714 (leukemia) to €4,645 (pancreas), and for palliative intent, from €938 (bone metastases) to €1753 (brain metastases). According to the technique used, costs ranged from €1,475 (3D conformal) to €3,608 (rotational IMRT), and by fractionation scheme, from €1,308 (extreme hypofractionation) to €4,094 (standard fractionation). Accounting for 2023 complexity levels, mean treatment cost rose by 0.9%, but varied widely by tumor site, with a 13% increase for stomach cancer, and decreases of −15.0, −24.4, and − 17.2% in myeloma, pancreas, and lung cancer, respectively. Including complementary examinations and hospital overhead costs, mean cost increased by 15.6%.

**Conclusion:**

This study provides a first approximation to EBRT cost using time-driven activity-based costing (TD-ABC) in Catalonia showing the feasibility of the assessment. For each indication, average treatment cost increases with the associated complexity. Additionally, costs decrease with hypofractionation schemes, largely due to lower equipment weight in treatment cost. Consequently, the adoption of stereotactic techniques is driving cost decreases. Overall, this model represents a robust tool for analyzing different possible scenarios, including changes in fractionation and complexity.

## 1 Introduction

Radiotherapy is a key strategy in multidisciplinary cancer care, whose importance in treatments of both radical and palliative intent has been reinforced by the clinical application of innovative technologies such as image-guided and stereotactic intensity-modulated radiotherapy (1). However, these new, more complex techniques, demanding a greater use of time and resources, have led to continuous cost increases (2, 3). In this context, the European Society for Radiotherapy-Health Economics in Radiation Oncology project (ESTRO-HERO) aimed to promote the use of evidence in resource planning in the field of radiation oncology. The costing of healthcare interventions is a necessary first step in the economic evaluations that support reimbursement decisions and policy formulation (4), but there is little evidence describing the costs in radiotherapy in a precise and detailed way (5). Thus, a model that provides evidence on the resource requirements and costs of current practices and technical advances in external beam radiotherapy (EBRT) was developed within the framework of the ESTRO-HERO project.

Using the time-driven activity-based costing (TD-ABC) methodology, a cost accounting model was developed to estimate the cost of EBRT from the perspective of national healthcare providers in Europe. The aim is to enable the assessment of the costs of different EBRT indications, treatments, and techniques as well as of national resource requirements and utilization in order to inform reimbursement decisions and resource planning.

Having completed the model validation stage at the European level, the objective of this study was to estimate the cost of EBRT in public health care centers in Catalonia (Spain), according to the ESTRO-HERO costing model for 2018, and to assess the feasibility of translating the EU model to a national/regional level. To this end, the Working Group on Costs of Radiation Oncology in Catalonia was created, involving professionals from different centers, representative of radiotherapy activity in Catalonia and the Department of Health Cancer Plan.

## 2 Materials and methods

### 2.1 ESTRO-HERO costing model

The cost estimation model developed by the ESTRO-HERO working group uses TD-ABC methodology, in which the cost is allocated according to the activity times related to the radiotherapy indications and techniques (6).

**Figure 1:**
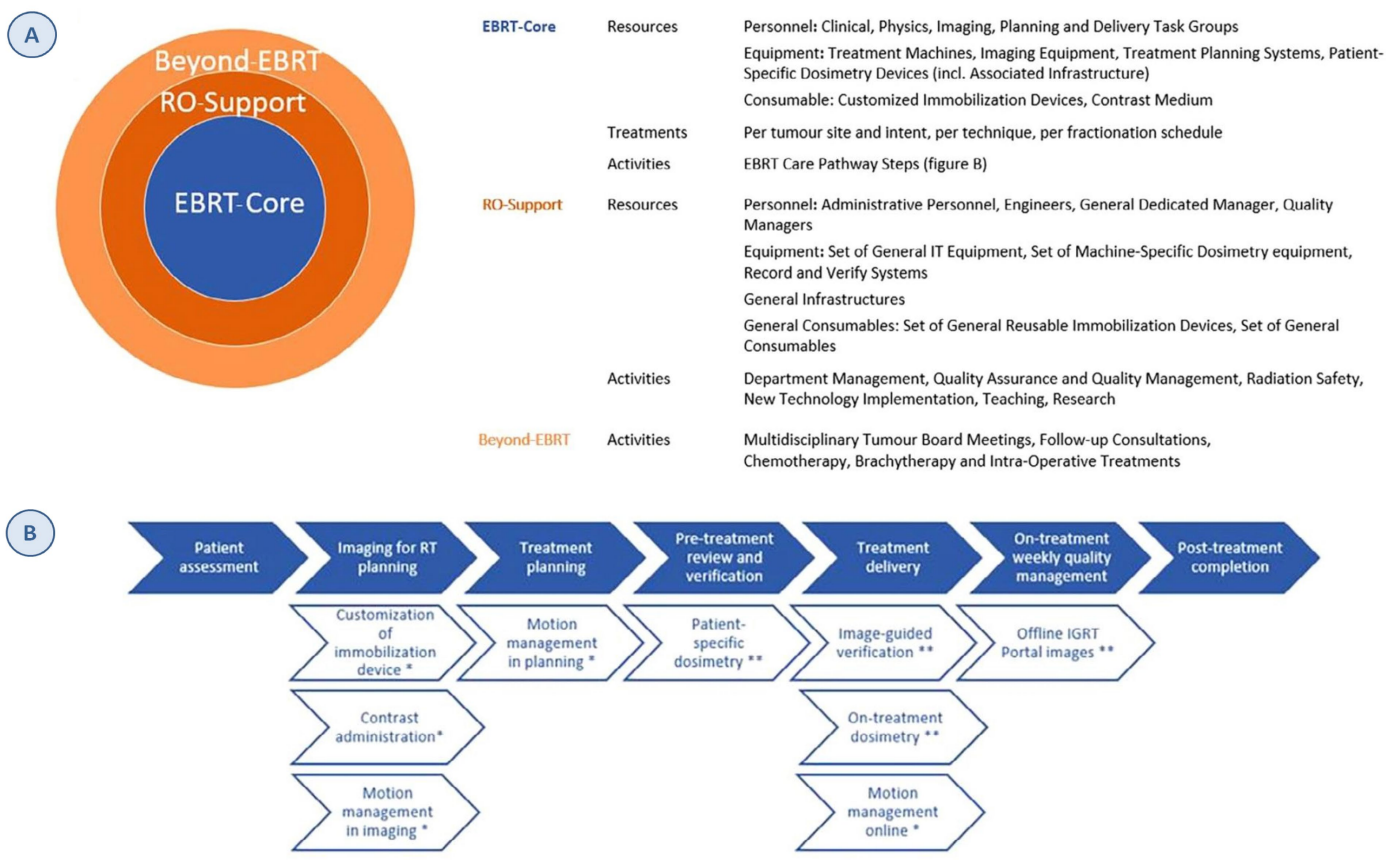
ESTRO-HERO model structure. (A) Inputs for each of the three components of the model. (B) EBRT care-pathway steps. Full arrows: standard activities. Open arrows: optional activities; *OPTIONAL activities defined by tumor site and intent, **optional activities defined based on the technique. Source: Defourny et al. (6). Reprinted with permission from https://www.sciencedirect.com/science/article/abs/pii/S0167814019329512 by Defourny et al. (6), licensed under CC-BY.

The structure of the model has three main components: (1) activities directly related to the administration of radiotherapy treatment (EBRT-Core), (2) necessary support activities (RO-Support) and (3) activities performed by RO personnel within the multidisciplinary oncology team, not related to EBRT as such (Beyond-EBRT) (Figure 1).

The first component has three subcategories: (1) *resources*: personnel, equipment (including its relevant infrastructure) and consumables; the personnel and equipment are grouped according to the task performed (planning, imaging, etc.); (2) *treatments*: the number of EBRT treatments by tumor location, treatment intent, technique, and fractionation scheme; and (3) *activities*: derived from the process map developed by the American Association of Physicists in Medicine (AAPM) (7). The process map structure was adapted to costing purpose of radiotherapy as it was originally developed for quality management. The time estimates also allow the differentiation of six different techniques, capturing the variability and complexity of the treatments (Figure 1).

The second component includes support activities for radiotherapy care, such as quality managers or dosimetry teams, and the third encompasses the activities carried out by RO personnel that are not directly related to the radiotherapy activity. In the cost allocation for these two components, 20% of the costs are assigned using the total number of treatments as the denominator and 80% of the costs using the number of fractions (8, 9).

### 2.2 Application of the model in Catalonia (Spain)

The public health care system in Catalonia has 11 radiotherapy centers covering a population of 7.5 million people (Supplementary Figure 1). For the application of the costing model, the following data from all 11 centers in 2018 were used: N departments, N personnel, equipment, N cases treated according to tumor location, treatment intent (curative/palliative), fractionation scheme, and technique (10). These variables were complemented by a data set for a hypothetical country “Europalia” used in the model validation, incorporating mean values calculated in a European context, which the working group adapted to the Catalan context.

*Resources* (Table 1). The calculation included full-time equivalent staff and annual salaries, including employer contributions to social security. The cost of the equipment accounted for depreciation and annual maintenance, including costs related to the infrastructure, commissioning personnel, and quality control checks.

**Table 1:** Resource input parameters for EBRT-core and RO-support.

Personnel	FTE^1^	Annual salary (€)^2^	Paid hours/day	Work days/week	Annual holidays (days)
**EBRT-core**					
Clinical task group					
RO consultants (dept head)	7.7	103,189	8.5	5	34
RO consultants	53.2	85,554	8.5	5	34
Nurses	48.0	45,607	7.5	5	34
Nursing assistants	16.0	29,869	7.5	5	34
RT technicians	15.6	33,768	7.5	5	34
Auxiliary room	5.5	27,440	7.5	5	34
Physics task group					
Physicists (dept. head)	6.4	103,189	8.5	5	34
Physicists	28.4	85,554	8.5	5	34
Imaging task group					
RO consultants (dept. head)	1.65	103,189	8.5	5	34
RO consultants	11.4	85,554	8.5	5	34
RT technicians	15.6	33,769	7.5	5	34
Planning task group					
Physicists (dept. head)	1.6	103,189	8.5	5	34
Physicists	7.1	85,554	8.5	5	34
Dosimetrists	45.0	33,769	7.5	5	34
RT Technicians	15.6	33,768	7.5	5	34
Delivery task group					
RO consultants (dept. head)	1.65	103,189	8.5	5	34
RO consultants	11.4	85,554	8.5	5	34
RT technicians	109.2	33,768	7.5	5	34
Auxiliary room	5.5	27,440	7.5	5	34
**RO-Support**					
Social workers/psychologists	6	45,607			
Quality managers	6	45,607			
Administrative personnel	40	33,461			

General consumables	Units^5^	Purchase price^6^	Maintenance contract	Lifetime (years)
**EBRT-core**				
Customized immobilization devices		50	–	–
Contrast medium (per unit)		44	–	–
**RO-support**				
Set of general consumables	11	72,500	0%	1
Set of general reusable immobilization devices	44	55,000	0%	5

Equipment and associated infrastructure	Units^3^	Purchase price^4^	Maintenance contract	Lifetime (years)	Annual quality control time/unit (days)	Commissioning time/unit (days)
**EBRT-core**						
Treatment machines						
Linear accelerator	33	1,976,043	10%	12	15	30
Dedicated stereotactic unit	1	3,500,000	10%	12	15	30
Associated bunkers	34	482,115	2%	30		
Imaging equipment						
CT simulator	10	347,464	10%	12	5	5
Associated bunkers	10	378,412	2%	30		
Treatment planning systems						
Treatment planning systems	11	231,076	10%	5	4	20
Associated planning room	11	378,412	2%	30		
Patient-specific dosimetry devices	11	10,000	10%	5	2	10
**RO-Support**						
General infrastructure	11	1,504,470	2%	30		
Set of general IT equipment	11	100,000	10%	5		
Set of machine-specific dosimetry equipment	11	10,000	10%	5		
Record and verify systems	11	110,441	10%	5	3	10

FTE, full-time equivalent; EBRT, external beam radiotherapy; RO, radiation oncology; CT, computer tomography; RT, radiotherapy; IT, information technology. ^1^Source: Rodríguez et al. (10) Infraestructura i equipament en el Sistema Nacional de Salud el període 2015–2020. Sociedad Española de Oncología Radioterápica (SEOR) 2018. ^2^Source: Collective working agreement in acute care hospitals, social health care centers, and mental health centers affiliated with the Catalan Health Service. Salary tables from level C career professionals; excludes bonus payments, but includes an estimated proportion for the on-call shifts. ^3^Source: Rodríguez et al. (10) Infraestructura i equipament en el Sistema Nacional de Salud el període 2015–2020. Sociedad Española de Oncología Radioterápica (SEOR) 2018. The equipment data are from 2015, but the number of machines listed in this report was updated to 2018. ^4^Source: Defourny et al. (6). ^5^Set of general consumables: one per department; set of general reusable immobilization devices: one per treatment and imaging machine. ^6^Source: expert estimates.

*Treatments* (Supplementary Table 1): N annual radiotherapy treatments, according to tumor location and treatment intent (20 indications with curative intent, organized by primary tumor, and 3 palliative indications, organized by metastatic location), technique (single-field radiotherapy, 2D radiotherapy [2D-RT], 3D conformal radiotherapy [3D-CRT], intensity-modulated radiotherapy [IMRT], rotational IMRT and stereotactic techniques), and N fractions administered.

*Activities* (Supplementary Table 2): Time used (in minutes) by staff and for equipment is defined by activity, differentiating six techniques. For Europalia, the HERO-WP3 expert panel estimated staff discrete time for each core activity (EBRT-Core) based on published evidence (11–13). The equipment costs incurred by the different activities were calculated based on the staff time dedicated to them. For activities involving different task groups working in parallel, the cost estimate is based only on the time used by the reference task group. When task groups work sequentially, time use across all the activities was taken into account. Staff time dedicated to support activities and activities indirectly related to RT (RO-Support and Beyond-EBRT) was defined by the HERO-WP3 panel based on usual practice. These parameters were adapted to the Catalan context by the radiation oncologists and medical physicists in the working group (Supplementary Table 3).

Brain radiosurgery, that is, single-session stereotactic radiosurgery, was not included in this analysis. Respiratory control maneuvers such as deep inspiration or gating were not considered separately, but extra time was added to respiratory control for each disease, especially in breast and lung cancer.

### 2.3 Incorporation of new techniques and fractionation schemes

Recent years have seen an increase in the complexity of the treatments and the use of shorter fractionation schemes, so in a second phase, the costs of the RT treatment were estimated, including the percentages of use of the fractioning techniques and schemes used in 2023 (Supplementary Table 4). The rest of the parameters remain constant; only the percentage of use of the fractionation techniques and schemes were changed.

### 2.4 Complementary estimates to the ESTRO-HERO model

The following complementary estimates were included: complementary examinations for planning, stereotactic body radiation therapy (SBRT) fiducial markers, and hospital overhead costs (14). The costs corresponding to the radiation oncology service/department were already included.

## 3 Results

**Figure 2:**
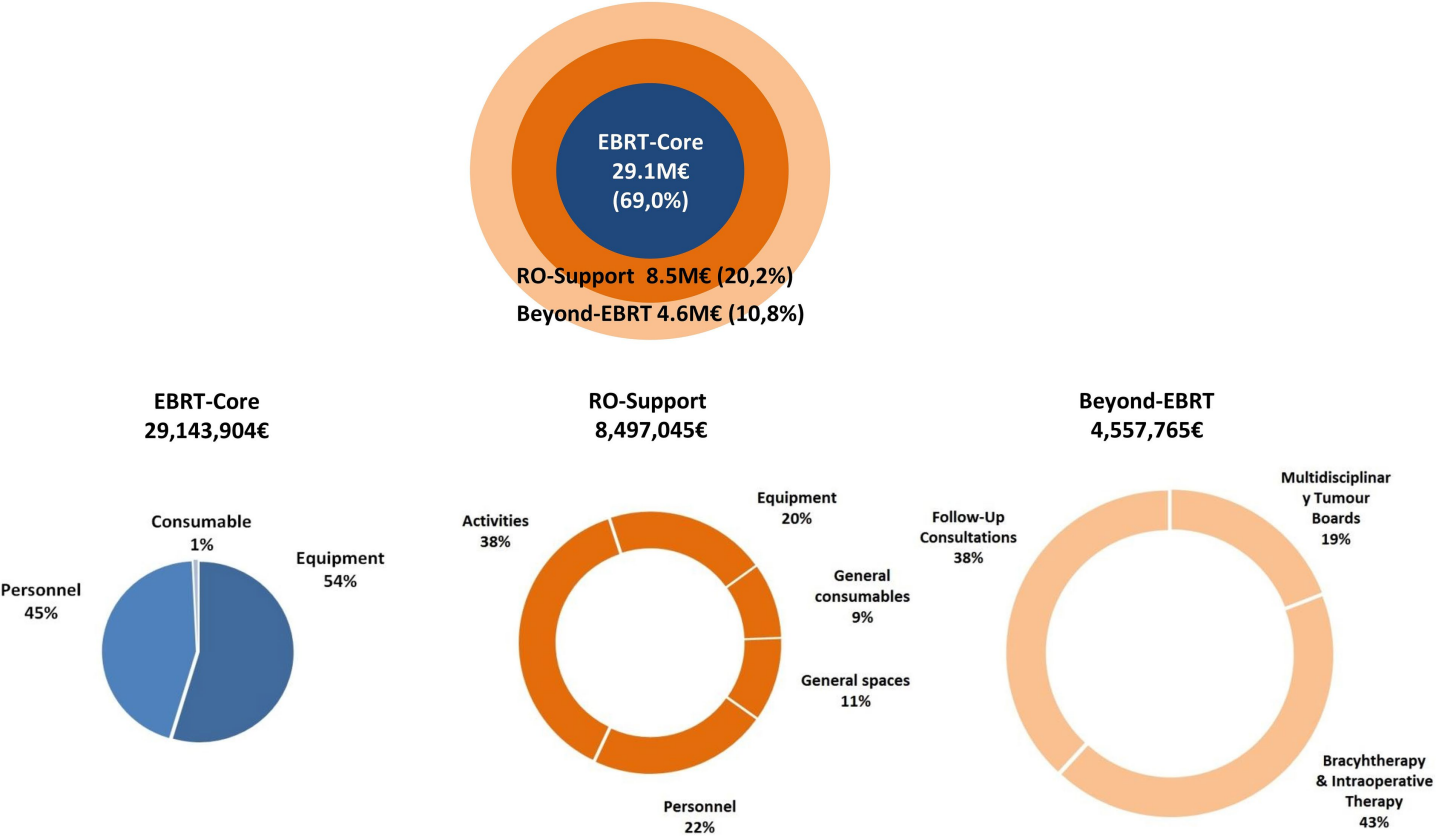
Proportional cost of EBRT-core, RO-support, and beyond-EBRT activities, 2018 (%).

The total cost of EBRT in 2018 was estimated at EUR 42.2 million. Activities directly related to the administration of treatment represented 69.0% of the total cost, while support activities and not directly related EBRT costs represented 20.2 and 10.8% of the total (Figure 2 and Supplementary Table 5).

The mean cost of radical treatment ranged from €1714 (leukemia) to €4,645 (pancreas), and with a palliative intent, from €938 (bone metastases) to €1753 (brain metastases) (Table 2). According to the technique used, costs ranged from €1,475 (3D conformal) to €3,608 (rotational IMRT), and by fractionation scheme, from €1,308 (extreme hypofractionation) to €4,094 (standard fractionation).

**Table 2:** Mean cost per external beam radiotherapy treatment, according to intent, technique, and fractionation schedule, 2018 (€).

	Courses (n)	EBRT cost (€)		
		Mean	Range	
**Overall**	14,138	2,985	731	5,257
**Curative intent**	10,484	3,586	1,276	5,257
Bladder	124	4,340	4,337	4,403
Brain	353	3,740	2,392	4,665
Breast	3,543	3,055	1883	5,102
Cervix	162	3,777	3,329	4,884
Head and neck	840	4,351	3,792	5,039
Leukemia	31	1714	1,639	1800
Lung	1,185	4,296	2,121	5,257
Lymphoma	318	2,279	1789	3,186
Melanoma	394	3,275	2,291	4,599
Myeloma	56	3,141	2,816	3,350
Esophagus	190	4,201	3,637	4,981
Pancreas	130	4,645	4,269	4,891
Prostate	1765	4,215	2,996	4,950
Rectum	803	3,075	1,276	4,325
Soft tissue	182	3,311	2,698	3,974
Stomach	75	3,269	2,698	3,951
Testis	13	2,262	1883	3,260
Thyroid	13	4,066	3,419	4,540
Uterus	230	3,435	3,124	3,701
Vagina	77	3,667	3,329	3,951
**Palliative intent**	3,654	1,259	731	3,510
Brain metastases	573	1753	1,158	2,250
Bone metastases	914	938	731	3,510
Lymph nodes metastases	2,167	1,264	731	2,101
Technique				
3D conformal RT	3,409	1,475	731	4,123
Intensity-modulated RT	4,536	3,326	865	5,257
Intensity-modulated rotational RT	5,989	3,608	874	4,862
Stereotactic techniques	204	2,326	2,121	3,510
Fractionation schedule				
Standard fractionation schedule (25 fractions)	7,242	4,094	1,639	5,257
Hypo fractionation schedule (11–24 fractions)	2,782	2,577	1883	3,350
Extreme hypo fractionation schedule (1–10 fractions)	4,113	1,308	731	3,510

RT, radiotherapy.

**Figure 3:**
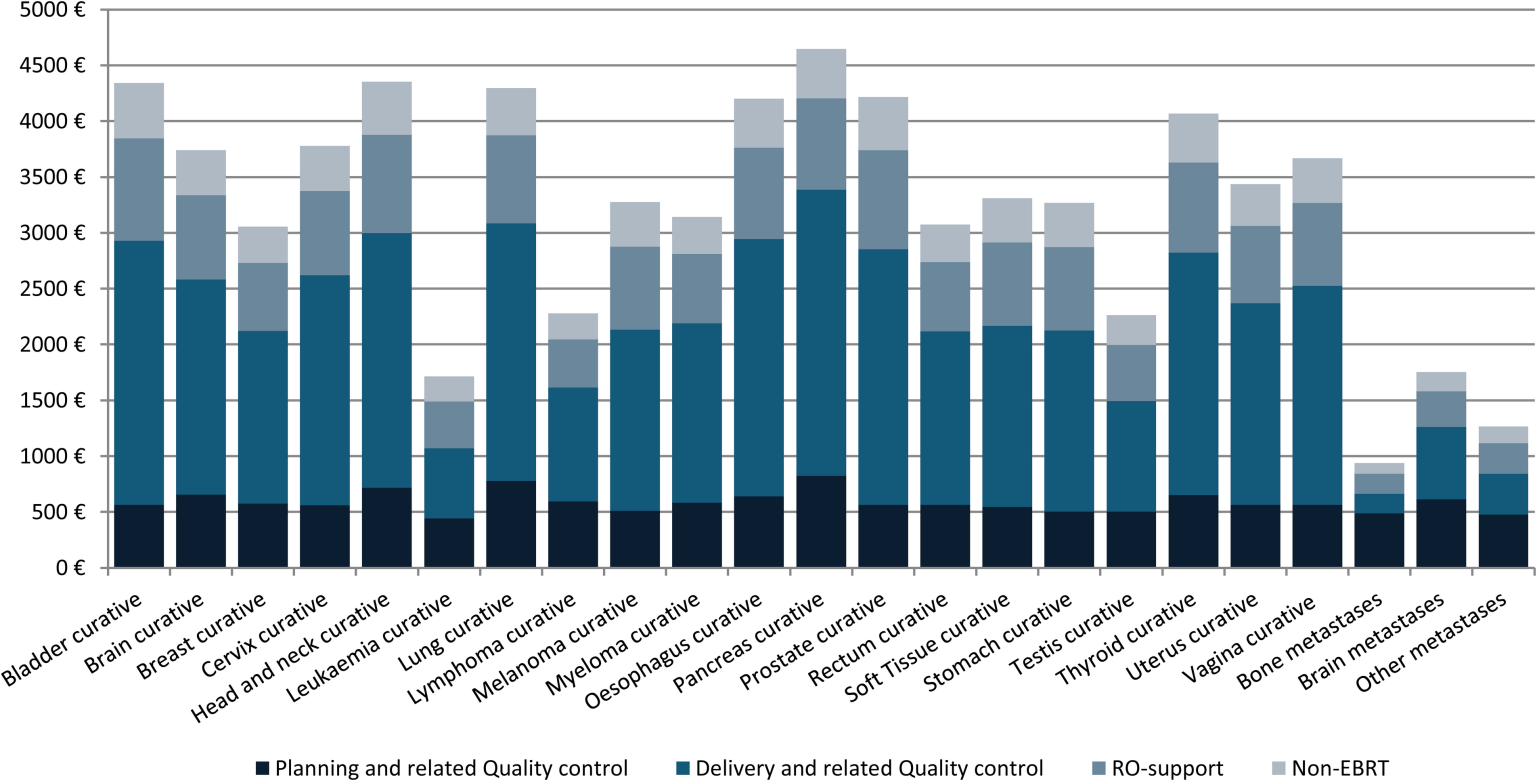
Average EBRT cost per treatment and activity, 2018 (€).

Figure 3 shows the mean cost of RT treatment according to tumor location and type of activity for the year 2018. The mean cost of RT treatment was also estimated according to tumor location, technique, fractionation scheme, and type of activity for all indications (Supplementary Table 6).

Taking into account the level of complexity in 2023, the mean cost of treatment rose by 0.9% overall, but it ranged widely according to tumor site, from a 13% rise in the case of stomach cancer, to a fall of −15.0, −24.4, and −17.2% in myeloma, pancreas, and lung cancer, respectively (Table 3; Supplementary Tables 7, 8). The mean cost of the treatment increased by 15.6% once complementary examinations and hospital overhead costs were included (Supplementary Table 9).

**Table 3:** Comparison of average EBRT cost per treatment, technique, and fractionation schedule in selected tumors by techniques and fractionation schedules 2018 and 2023.

	Fractionation schedule	Average EBRT treatment cost (€)						
		% Techniques and fractionation schedules 2018			% Techniques and fractionation schedules 2023			Variation 2018–23 (%)
		Courses (*n*)	%	Mean cost (€)	Courses (*n*)	%	Mean cost (€)	
**Bladder**		124	100.0	4,340	124	100.0	4,524	4.2
IMRT rotational	Standard	124	100.0	4,340	124	100.0	4,524	4.2
**Breast**		3,543	100.0	3,055	3,543	100.0	3,192	4.5
3D-CRT	Standard	124	3.5	3,206	213	6.0	3,632	13.3
3D-CRT	Hypofractionation	230	6.5	2039	496	14.0	2,342	14.8
IMRT	Standard	744	21.0	4,202	531	15.0	4,559	8.5
IMRT	Hypofractionation	1,382	39.0	2,628	1,240	35.0	2,908	10.7
IMRT rotational	Standard	372	10.5	3,959	319	9.0	4,187	5.8
IMRT rotational	Hypofractionation	691	19.5	2,497	744	21.0	2,702	8.2
**Head and neck**		840	100.0	4,351	840	100.0	4,509	3.6
IMRT	Standard	84	10.0	4,592	84	10.0	4,887	6.4
IMRT rotational	Standard	756	90.0	4,325	756	90.0	4,467	3.3
**Lung**		1,185	100.0	4,296	1,185	100.0	3,558	−17.2
3D-CRT	Standard	—	—	—	356	30.0	3,939	
IMRT	Standard	563	47.5	4,519	142	12.0	4,906	8.6
IMRT rotational	Standard	563	47.5	4,275	213	18.0	4,516	5.6
Stereotactic techniques		59	5.0	2,377	474	40.0	2,437	2.5
**Myeloma**		56	100.0	3,141	56	100.0	2,670	−15.0
3D-CRT	Standard	—	—	—	56	100.0	2,670	
IMRT	Standard	39	70.0	3,194	—	—	—	
IMRT rotational	Standard	17	30.0	3,019	—	—	—	
**Esophagus**		190	100.0	4,201	190	100.0	4,223	0.5
IMRT	Standard	133	70.0	4,274	—	—	—	
IMRT rotational	Standard	57	30.0	4,029	190	100.0	4,223	4.8
**Pancreas**		130	100.0	4,645	130	100.0	3,509	−24.4
3D-CRT	Standard	—	–	–	23	17.5	4,098	
IMRT	Standard	65	50.0	4,767	10	7.5	5,055	6.0
IMRT rotational	Standard	65	50.0	4,522	33	25.0	4,670	3.3
Stereotactic techniques		–	–	–	65	50.0	2,491	
**Prostate**		1765	100.0	4,215	1765	100.0	4,422	4.9
IMRT	Standard	124	7.0	4,740	124	7.0	5,115	7.9
IMRT rotational	Standard	1,465	83.0	4,317	1,465	83.0	4,518	4.7
IMRT rotational	Hypofractionation	177	10.0	3,000	177	10.0	3,132	4.4
**Rectum**		803	100.0	3,075	803	100.0	3,189	3.7
IMRT	Standard	169	21.0	3,983	56	7.0	4,290	7.7
IMRT	Extreme hypofractionation	72	9.0	1,368	24	3.0	1,453	6.2
IMRT rotational	Standard	393	49.0	3,750	506	63.0	3,922	4.6
IMRT rotational	Extreme hypofractionation	169	21.0	1,324	217	27.0	1,385	4.5
**Stomach**		75	100.0	3,269	75	100.0	3,693	13.0
3D-CRT	Standard	38	50.0	2,871	19	25.0	3,215	12.0
IMRT	Standard	19	25.0	3,777	19	25.0	4,082	8.1
IMRT rotational	Standard	19	25.0	3,558	38	50.0	3,738	5.1

3D-CRT, 3-dimensional conformal RT; EBRT, external beam radiotherapy; IMRT, intensity-modulated RT; IMRT rotational, intensity-modulated rotational RT.

## 4 Discussion

The European Society for Radiotherapy and Oncology - Health Economics in Radiation Oncology project (ESTRO-HERO) developed a cost accounting model to estimate the cost of external beam radiotherapy (EBRT) treatment from the perspective of healthcare providers at a national level in Europe, using the time-driven activity-based costing methodology. Subsequently, a working group on costs of radiation oncology, created under the auspices of the Department of Health Cancer Plan, applied the ESTRO-HERO model to estimate the cost of EBRT in Catalonia. The working group was made up of expert radiation oncologists and medical physicists from different centers, representative of radiotherapy activity in Catalonia and under the Cancer Plan.

The ESTRO-HERO model comprises three components: the first is directly related to the administration of external radiotherapy, the second encompasses support activities, and the third accounts for the activities carried out by RO personnel that are not directly related to the radiotherapy activity in the context of the multidisciplinary oncology teams. As seen in Figure 2, the costs derived from the first component—related to administering EBRT—have the greatest weight in the total cost. The overall mean cost of treatment with a curative intent was €3,586, ranging from €1714 to €4,645 depending on the tumor site (Table 2). These figures are lower than for other cancer treatments, demonstrating once again that despite the upfront investment required, radiotherapy continues to be very cost-effective.

Regarding the mean cost of treatment by primary tumor site, results show that for the five tumors with the highest incidence (breast, prostate, lung, head and neck, and rectum), the average cost ranges from €3,055 for breast cancer to €4,351 for tumors of the head and neck. These differences are a direct result of the widespread use of hypofractionated schemes for breast cancer, and the negligible use of these techniques for head and neck cancer. As for the rest of the tumors, the highest treatment costs were for pancreatic cancers (€4,645), which is consistent with the use of classic fractionation schemes and the great technical complexity.

The technique-specific results are unsurprising (Table 2). The 3D techniques have a mean cost of €1,475, and this increases with complexity, reaching €3,326 for IMRT and €3,608 for rotational IMRT. The apparently low cost of stereotactic techniques is somewhat unexpected (€2,326 euros), but as the table shows, the cost of treatment with classic fractionation is €4,094; with hypofractionation, €2,577; and with extreme hypofractionation, €1,308, and stereotactic techniques are always used with extreme hypofractionation schemes. Likewise, most of the costs, across all diseases, are associated with the administration of the treatment (Figure 3), which explains why administering fewer fractions has such an important impact on the overall cost. This result is also found in a multicenter time-driven activity-based costing study in Belgium conducted by the Belgian Health Care Knowledge Centre, as well as in another study analyzing the financial impact of SBRT for oligometastatic disease in Belgium (8, 15). The weak relationship between treatment complexity and cost is also partly due to the widespread use of simulation units, treatment planning systems, and in general, technology that allows performing techniques of any complexity. Extreme hypofractionation requires a high-performance linear accelerator that incorporates an excellent image-guided RT system, rotational IMRT, a 6-degree-of-freedom table, etc. While such a complete accelerator is not necessary for a classically fractionated treatment, the upgrading of equipment carried out in recent years in Catalonia has afforded the region a park of technologically advanced accelerators that can be used for any treatment, regardless of complexity.

The average costs of treatments with a palliative intent are lower than for radical ones. However, some treatments that are considered palliative can be relatively expensive, as evidenced from the higher end of the ranges seen in Table 2. This elevated cost is due to the use of complex techniques such as hippocampal preservation in palliative cerebral irradiation, which has shown a positive impact on cognition, or the use of SBRT techniques in some bone metastases. Thus, it is important to base any increase in complexity on evidence; otherwise it can increase costs without benefitting patients.

Taking into account the change complexity of treatments delivered in 2023, there was a notable decrease in costs for pancreatic and lung cancer. This decrease can be attributed to the adoption of hypofractionated techniques, which generally reduce the cost despite increasing the complexity, for example from using respiratory control systems (Table 3). When comparing the results of 2018 and 2023 in relation to the indication and the technique used, the greater weight of fractionation compared to complexity in the cost of treatment is clear.

Various studies have estimated the costs of external radiotherapy in different contexts and with different methodologies (2, 5, 8, 14–20). Our results show significantly lower costs than those calculated in studies using activity-based costing. This is mainly due to the difference in salaries of health professionals between European countries (21). Compared to the studies by Defourny et al. (6, 18) conducted in Europalia (a hypothetical country utilized in the validation of the HERO cost model, incorporating mean values calculated within a European context) and Belgium, the salaries of Catalonia’s clinical task force are 49 and 66% lower, respectively. Similarly, the salaries of the physics task force in Catalonia are between 4 and 17% lower, and the salaries of the imaging task group are between 31 and 54% lower. On the other hand, the estimates of equipment costs are similar. Alternatively, it is important to highlight that, despite the fact that the costs are lower, the proportional cost of SBRT compared to longer schedules in Catalonia is similar to that found in the study of Nevens et al. (15). Using a microcosting methodology, the estimated cost of RT in rectal cancer in the study by Hanly et al. (19) was similar to ours; however, this was not the case in the study by Perrier et al. (20) for head and neck cancers, where the estimated cost was much lower.

One limitation of the study is that the cost model used may lead to an underestimation of the resources and costs of EBRT at the regional or national level, since the hospital overhead costs are not included, only those corresponding to the departments (6). Although there is some variability in relation to the weight of this type of cost over the total, in this study we made a complementary estimate of the overhead costs based on a recent study carried out by Spencer et al. (14) in the UK.

In the context of personalized radiotherapy in the era of precision medicine, one of the most imminent developments is the systemic deployment of adaptive techniques during the course of treatment (22), which will substantially increase the cost of activities directly related to treatment administration. Process automation tools, including those based on artificial intelligence, can mitigate this extra cost by reducing the time required of staff and for the treatment itself, but their adoption will in turn mean a new expense to be taken into account (23).

On the other hand, this costing study shows that, in our setting, the most important contributor to the cost of EBRT is the occupation of the treatment unit, that is, the number of sessions, hence the tendency to try to hypofractionate as much as possible. Moreover, this modality is also more comfortable for patients, and it also helps reduce waiting lists. However, hypofractionation schemes should mainly be applied with an eye toward minimizing toxicity in healthy tissue, ensuring that the patient’s overall quality of life is improved.

Both adaptive radiotherapy (and especially the set of tools that it involves) and the increasing use of hypofractionation in most tumor sites will entail changes in the roles of radiation oncologists, radiotherapy technicians and medical physicists. Professionals will have to adapt to a changing environment, without forgetting to center the patient and their needs, adopting appropriate measures to ultimately enhance their quality of life. Additional studies will be required to assess the costs in this new environment.

Finally, the application of the HERO costing model can support health care cancer policy and decision-making in Catalonia. Firstly, it promotes the utilization of evidence in resource planning in the field of radiation oncology. Secondly, the TD-ABC methodology contributes significantly by streamlining and optimizing the entire care cycle in radiotherapy. By accurately mapping and analyzing each step of the process, TD-ABC helps identify inefficiencies, reduce costs, and improve resource allocation, ultimately enhancing patient outcomes and care quality throughout the radiotherapy treatment pathway (24). And, finally, by enabling the costing of radiotherapy treatments at a regional level, it provides support for reimbursement decisions and policy formulation (25). This approach allows policymakers to better understand the financial implications of radiotherapy services, promoting a sustainable allocation of healthcare funds. Additionally, it aids in developing standardized reimbursement rates that reflect the actual costs of providing high-quality care, ensuring that financial resources are allocated in a way that supports both efficiency and accessibility. This data-driven approach not only guides reimbursement strategies but also facilitates long-term planning for radiotherapy infrastructure and workforce development, ultimately improving access to cancer care across the region.

In conclusion, this study is a first approximation to the cost of external beam radiotherapy using time-driven activity-based costing (TD-ABC) in Catalonia. For each indication, the average cost per treatment increases with the associated complexity. Moreover, costs decrease when hypofractionation schemes are used, mainly due to the lower weight of the equipment in the treatment cost. Consequently, the adoption of stereotactic techniques is driving cost decreases. All in all, this model represents a powerful tool to analyze different possible scenarios, such as changes in fractionation and complexity.

## Data Availability

The original contributions presented in the study are included in the article/supplementary material, further inquiries can be directed to the corresponding author.
