# Supplementary material for: Challenges in assessing national radiotherapy costs: application of the ESTRO-HERO model in Spain
Source: Front Public Health. 2024 Dec 19;12:1474376. doi: 10.3389/fpubh.2024.1474376 (PMC11694224; doi:10.3389/fpubh.2024.1474376)
Supplement: Supplementary file 1 [file Data_Sheet_1.docx]

Supplementary Material

# Supplementary Figures and Tables

## Supplementary Figures

**Supplementary Figure 1.** Map of Catalonia showing RT centers distribution.


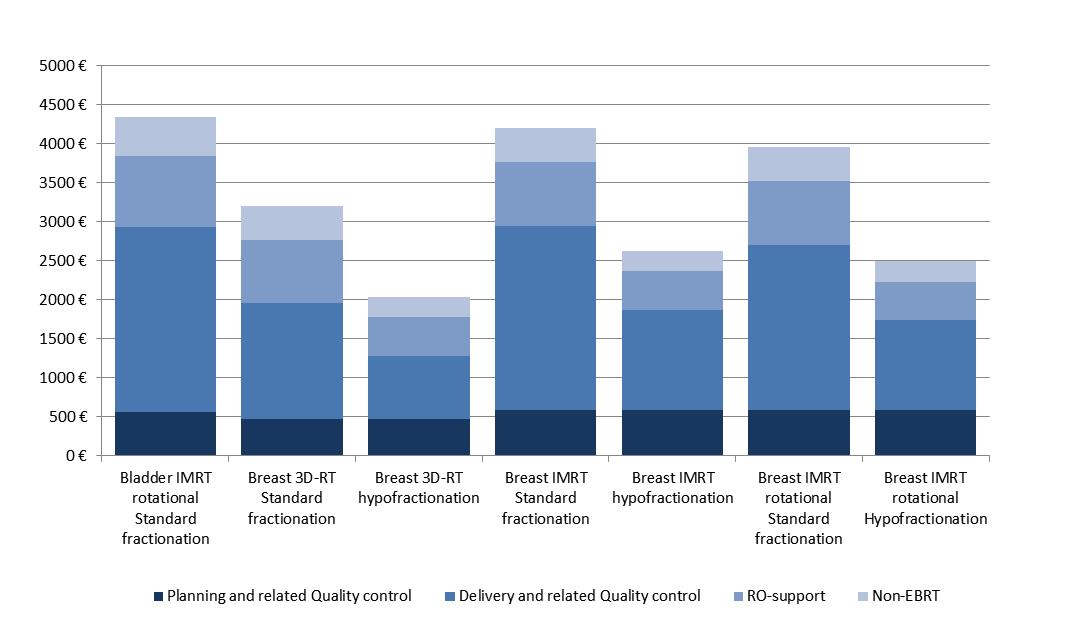


**Figure 2a.** Average EBRT cost per treatment, technique, fractionation schedule, and activity in selected tumors, 2018 (€)


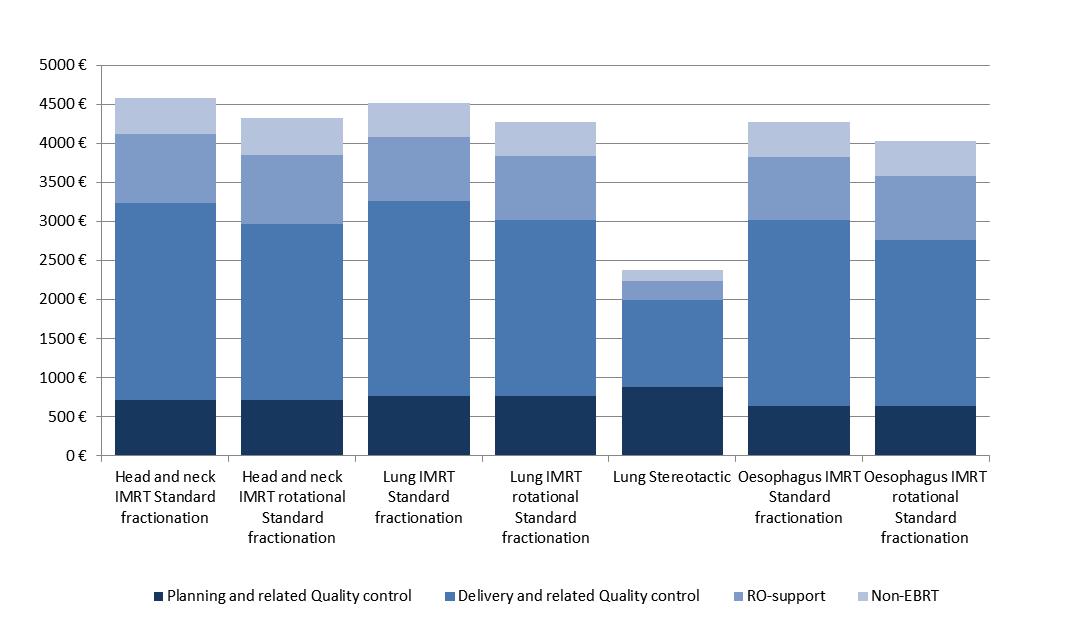


**Figure 2b.** Average EBRT cost per treatment, technique, fractionation schedule, and activity in selected tumors, 2018 (€)

**
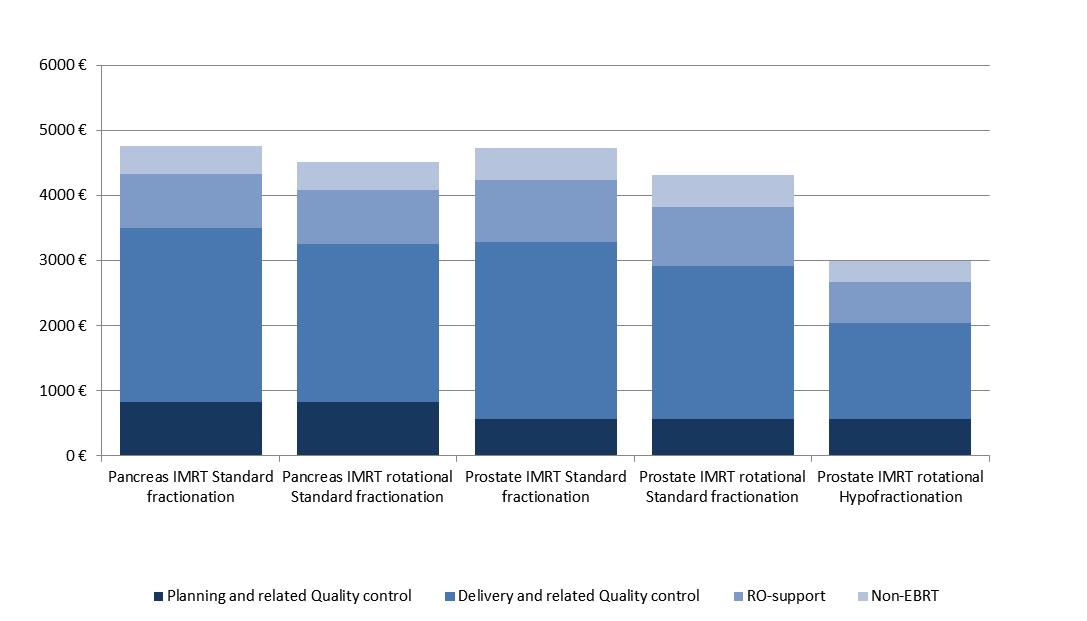
**

**Figure 2c.** Average EBRT cost per treatment, technique, fractionation schedule, and activity in selected tumors, 2018 (€)


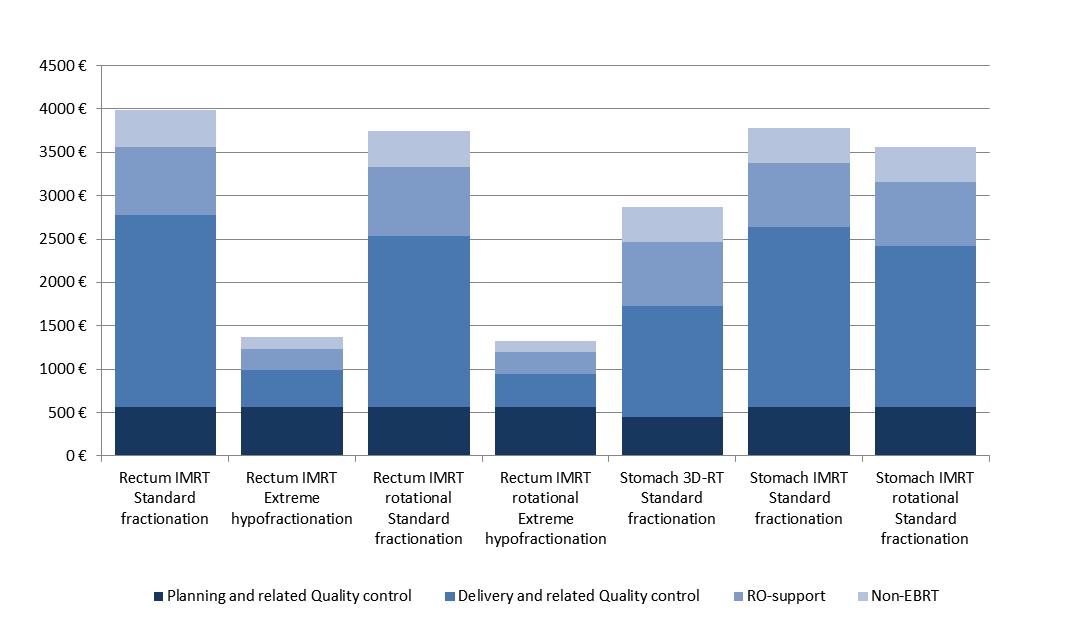


**Figure 2d.** Average EBRT cost per treatment, technique, fractionation schedule, and activity in selected tumors, 2018 (€)

## Supplementary Tables

**Supplementary Table 1.** Inputs related to external beam radiotherapy treatment courses, 2018

| **Tumor site** | **Courses (n)** | **N fractions** | **Fractionation schedule** | | **%** | **3D-CRT** | | **IMRT** | **Rotational IMRT** | **Stereotactic** |
| --- | --- | --- | --- | --- | --- | --- | --- | --- | --- | --- |
| **Bladder** | 124 | 32 | Standard | | 100.0% |  | |  | 100.0% |  |
| **Brain** | 353 | 28 | Standard | | 5.0% |  | | 20.0% | 80.0% |  |
|  |  | 30 | Standard | | 65.0% |  | | 20.0% | 80.0% |  |
|  |  | 15 | Hypofractionation | | 30.0% |  | | 20.0% | 80.0% |  |
| **Breast** | 3.543 | 25 | Standard | | 15.0% | 10.0% | | 60.0% | 30.0% |  |
|  |  | 30 | Standard | | 20.0% | 10.0% | | 60.0% | 30.0% |  |
|  |  | 15 | Hypofractionation | | 65.0% | 10.0% | | 60.0% | 30.0% |  |
| **Cervix** | 162 | 25 | Standard | | 95.0% |  | | 80.0% | 20.0% |  |
|  |  | 33 | Standard | | 5.0% |  | | 80.0% | 20.0% |  |
| **Head and neck** | 840 | 28 | Standard | | 50.0% |  | | 10.0% | 90.0% |  |
|  |  | 33 | Standard | | 50.0% |  | | 10.0% | 90.0% |  |
| **Leukemia** | 31 | 12 | Hypofractionation | | 100.0% | 100.0% | |  |  |  |
| **Lung** | 1,185 | 25 | Standard | | 40.0% |  | | 50.0% | 50.0% |  |
|  |  | 30 | Standard | | 55.0% |  | | 50.0% | 50.0% |  |
|  |  | 5 | Extreme hypofractionation | | 5.0% |  | |  |  | 100.0% |
| **Lymphoma** | 318 | 15 | Hypofractionation | | 50.0% |  | | 70.0% | 30.0% |  |
|  |  | 10 | Extreme hypofractionation | | 50.0% |  | | 70.0% | 30.0% |  |
| **Melanoma** | 394 | 30 | Standard | | 50.0% | 50.0% | | 25.0% | 25.0% |  |
|  |  | 20 | Hypofractionation | | 50.0% | 50.0% | | 25.0% | 25.0% |  |
| **Myeloma** | 56 | 20 | Hypofractionation | | 100.0% |  | | 70.0% | 30.0% |  |
| **Esophagus** | 190 | 28 | Standard | | 100.0% |  | | 70.0% | 30.0% |  |
| **Pancreas** | 130 | 28 | Standard | | 100.0% |  | | 50.0% | 50.0% |  |
| **Prostate** | 1765 | 28 | Standard | | 20.0% |  | |  | 100.0% |  |
|  |  | 33 | Standard | | 70.0% |  | | 10.0% | 90.0% |  |
|  |  | 20 | Hypofractionation | | 10.0% |  |  | | 100.0% |  |
|  |  |  | |  |  |  |  | |  |  |
| **Rectum** | 803 | 25 | | Standard | 30.0% |  | 30.0% | | 70.0% |  |
|  |  | 28 | | Standard | 40.0% |  | 30.0% | | 70.0% |  |
|  |  | 5 | | Extreme hypofractionation | 30.0% |  | 30.0% | | 70.0% |  |
| **Soft tissue** | 182 | 25 | | Standard | 100.0% | 50.0% | 25.0% | | 25.0% |  |
| **Stomach** | 75 | 25 | | Standard | 100.0% | 50.0% | 25.0% | | 25.0% |  |
| **Testis** | 13 | 15 | | Hypofractionation | 96.0% | 50.0% | 25.0% | | 25.0% |  |
|  |  | 20 | | Hypofractionation | 4.0% | 50.0% | 25.0% | | 25.0% |  |
| **Thyroid** | 13 | 25 | | Standard | 26.0% |  | 50.0% | | 50.0% |  |
|  |  | 28 | | Standard | 30.0% |  | 50.0% | | 50.0% |  |
|  |  | 29 | | Standard | 44.0% |  | 50.0% | | 50.0% |  |
| **Uterus** | 230 | 23 | | Standard | 100.0% |  | 50.0% | | 50.0% |  |
| **Vagina** | 77 | 25 | | Standard | 100.0% |  | 50.0% | | 50.0% |  |
| **Brain metastases** | 573 | 5 | | Extreme hypofractionation | 40.0% | 10.0% |  | | 30.0% |  |
|  |  | 10 | | Extreme hypofractionation | 60.0% | 90.0% |  | | 10.0% |  |
| **Bone metastases** | 914 | 1 | | Extreme hypofractionation | 85.0% | 70.0% | 20.0% | | 10.0% |  |
|  |  | 10 | | Extreme hypofractionation | 15.0% | 60.0% | 30.0% | | 5.0% |  |
| **Other metastases** | 2,167 | 1 | | Extreme hypofractionation | 20.0% | 80.0% | 10.0% | | 10.0% |  |
|  |  | 5 | | Extreme hypofractionation | 40.0% | 80.0% | 10.0% | | 10.0% |  |
|  |  | 10 | | Extreme hypofractionation | 40.0% | 80.0% | 10.0% | | 10.0% |  |

2D-RT: 2D radiotherapy, 3D-CRT: 3-dimensional conformal RT, EBRT: external beam radiotherapy, IMRT: intensity-modulated RT, rotational IMRT: intensity-modulated rotational RT

*No data available on single-field RT or 2D-RT.

**Supplementary Table 2.** Time (in minutes) and resource inputs for the different steps in the external beam radiotherapy care-pathway

| **EBRT care-pathway steps and**  **optioal steps** | | **Personnel involved** | **Techniques** | | | | | | **Related equipment^1^** | **Possible Consumables^2^** |
| --- | --- | --- | --- | --- | --- | --- | --- | --- | --- | --- |
|  |  |  | **Single**  **field RT** | **2D-RT** | **3D-CRT** | **IMRT** | **IMRT Rotational** | **Stereo-tactic** |  |  |
| **Patient assessment** | | Clinical | 60 | 60 | 60 | 60 | 60 | 60 | - | - |
| **Imaging for radiotherapy planning ^3^** | | Clinical | 10 | 10 | 10 | 10 | 10 | 10 | - | - |
|  | | Imaging | 30 | 30 | 30 | 30 | 30 | 30 | Imaging machine | - |
|  | Customization of immobilization device ^3^ | Imaging | 10 | 10 | 10 | 10 | 10 | 20 | Imaging machine | Mask |
|  | Contrast administration ^3^ | Clinical | 10 | 10 | 10 | 10 | 10 | 10 | Imaging machine | Contrast |
|  | Motion management ^3^ | Imaging | 0 | 0 | 30 | 30 | 30 | 30 | Imaging machine | - |
| **Treatment planning** | | Clinical | 0 | 0 | 40 | 40 | 40 | 50 | TPS | - |
|  | | Physics | 15 | 15 | 20 | 20 | 20 | 115 | TPS | - |
|  | | Planning | 20 | 30 | 145 | 175 | 175 | 110 | TPS | - |
|  | Motion management | Clinical | 0 | 0 | 10 | 10 | 10 | 10 | TPS | - |
|  | | Planning | 0 | 0 | 60 | 60 | 60 | 60 | TPS | - |
| **Pre-treatment review and verification** | | Clinical | 5 | 5 | 15 | 15 | 15 | 15 | TPS | - |
|  | | Physics | 5 | 5 | 15 | 15 | 15 | 15 | TPS | - |
|  | | Planning | 5 | 5 | 15 | 15 | 15 | 15 | TPS | - |
|  | Patient-specific dosimetry | Physics | 10 | 10 | 30 | 30 | 30 | 30 | Patient-specific dosimetry devices | - |
| **Treatment delivery timeslot (excl. IGRT, In vivo, Motion management) (per fraction)^4^** | | Clinical | 0 | 0 | 0 | 5 | 5 | 30 | Treatment machine | - |
|  | | Delivery | 10 | 10 | 15 | 20 | 15 | 60 | Treatment machine | - |
|  | Image-guided verification ^4^ | Clinical | 5 | 0 | 3 | 10 | 10 | 10 | Treatment machine | - |
|  |  | Delivery | 5 | 5 | 5 | 5 | 5 | 10 | Treatment machine | - |
|  | On-treatment dosimetry ^4^ | Delivery | 5 | 5 | 5 | 0 | 0 | 0 | Treatment machine | - |
|  | Motion management ^4^ | Delivery | 0 | 0 | 5 | 5 | 5 | 5 | Treatment machine | - |
| **On-treatment weekly quality management** | | Clinical | 15 | 15 | 15 | 15 | 15 | 15 | - | - |
|  | | Imaging | 5 | 5 | 5 | 5 | 5 | 10 | - | - |
|  | | Delivery | 10 | 10 | 10 | 10 | 10 | 10 | - | - |
|  | Offline IGRT portal images | Clinical | 5 | 5 | 5 | 5 | 5 | 5 | - | - |
| **Post treatment completion** | | Clinical | 30 | 30 | 30 | 30 | 30 | 30 | - | - |
|  | | Delivery | 15 | 15 | 15 | 15 | 15 | 15 | - | - |

2D-RT: 2D radiotherapy, 3D-CRT: 3D-conformal radiotherapy, EBRT: external beam radiotherapy, IMRT: intensity-modulated radiotherapy, TPS: Treatment Planning System, IGRT: image guided radiotherapy

^1^ Equipment: time of equipment occupation is assumed equal to the time required of involved personnel, as the machines cannot operate on their own. This means that the results show the total time of personnel using the equipment if the tasks are performed sequentially (e.g. treatment planning), or the time used by a reference task group if tasks are performed in parallel (e.g. contrast administration by imaging task group). Sequential tasks are indicated by the repetition of the equipment for each personnel type; for parallel tasks, the equipment is shown only once next to the reference task group.

^2^ Consumables: These resources are directly traceable to specific EBRT courses and do not require estimation via the TD-ABC method; the number of courses requiring these consumables depend on tumor type and intent (data not shown).

^3^ We assume 2 people from the treatment delivery task group per shift on the imaging machine, hence times of involved personnel presented are multiplied by 2.

^4^ We assume 3 people from the treatment delivery task group per shift on the treatment delivery machine, hence times of involved personnel presented are multiplied by 3.

**Supplementary Table 3.** Distribution of personnel resource input parameters across EBRT-Core, RO-Support and Beyond-EBRT activities

| **Activities/Personnel resources** | | **Clinical**  **task group** | **Physics**  **task group** | **Imaging**  **task group** | **Planning**  **task group** | **Delivery**  **task group** |
| --- | --- | --- | --- | --- | --- | --- |
| **EBRT-Core** | Care-pathway activities | 35% | 52% | 88.5% | 88.5% | 88.5% |
| **RO-Support** | Departmental management & team meetings | 10% | 10% | 1% | 1% | 1% |
|  | Quality assurance & quality management (excl. annual machine-related quality control, breakdowns) | 2% | 2% | 1% | 1% | 1% |
|  | Radiation safety & radiation protection | 1% | 10% | 1% | 1% | 1% |
|  | Technology & techniques implementation | 3% | 10% | 1% | 1% | 1% |
|  | Teaching (academic & on-the-job training) | 2% | 2% | - | - | - |
|  | Research | 2% | 2% | - | - | - |
| **Beyond-EBRT** | Chemotherapy | - | - | - | - | - |
|  | Brachytherapy & intraoperative therapy | 10% | 12% | 7.5% | 7.5% | 7.5% |
|  | Follow-up consultation | 20% | - | - | - | - |
|  | Multidisciplinary tumor boards | 10% | - | - | - | - |

EBRT: external beam radiotherapy, RO: radiation oncology

**Supplementary Table 4.** Inputs for EBRT treatment courses, 2023

| **Tumor site** | **N fractions** | **Fractionation schedule** | **%** | **Single field RT** | **2D-RT** | **3D-CRT** | **IMRT** | **Rotational IMRT** | **Stereotactic** |
| --- | --- | --- | --- | --- | --- | --- | --- | --- | --- |
| **Bladder** | 32 | Standard | 100.0% |  |  |  |  | 100.0% |  |
| **Brain** | 28 | Standard | 5.0% |  |  |  |  | 100.0% |  |
|  | 30 | Standard | 65.0% |  |  |  |  | 100.0% |  |
|  | 15 | Hypofractionation | 30.0% |  |  |  |  | 100.0% |  |
| **Breast** | 25 | Standard | 10.0% |  |  | 20.0% | 50.0% | 30.0% |  |
|  | 28 | Standard | 20.0% |  |  | 20.0% | 50.0% | 30.0% |  |
|  | 15 | Hypofractionation | 70.0% |  |  | 20.0% | 50.0% | 30.0% |  |
| **Cervix** | 25 | Standard | 95.0% |  |  |  | 10.0% | 90.0% |  |
|  | 33 | Standard | 5.0% |  |  |  | 10.0% | 90.0% |  |
| **Head and neck** | 28 | Standard | 50.0% |  |  |  | 10.0% | 90.0% |  |
|  | 35 | Standard | 50.0% |  |  |  | 10.0% | 90.0% |  |
| **Leukemia** | 12 | Hypofractionation | 100.0% |  |  | 100.0% |  |  |  |
| **Lung** | 25 | Standard | 20.0% |  |  | 50.0% | 20.0% | 30.0% |  |
|  | 30 | Standard | 40.0% |  |  | 50.0% | 20.0% | 30.0% |  |
|  | 5 | Extreme hypofractionation | 40.0% |  |  |  |  |  | 100.0% |
| **Lymphoma** | 15 | Hypofractionation | 50.0% |  |  | 25.0% | 15.0% | 60.0% |  |
|  | 10 | Extreme hypofractionation | 50.0% |  |  | 25.0% | 15.0% | 60.0% |  |
| **Melanoma** | 30 | Standard | 50.0% |  |  | 50.0% |  | 50.0% |  |
|  | 20 | Hypofractionation | 50.0% |  |  | 50.0% |  | 50.0% |  |
| **Myeloma** | 20 | Hypofractionation | 100.0% |  |  | 100.0% |  |  |  |
| **Esophagus** | 28 | Standard | 100.0% |  |  |  |  | 100.0% |  |
| **Pancreas** | 28 | Standard | 50.0% |  |  | 35.0% | 15.0% | 50.0% |  |
|  | 5 | Extreme hypofractionation | 50.0% |  |  |  |  |  | 100.0% |
| **Prostate** | 28 | Standard | 20.0% |  |  |  |  | 100.0% |  |
|  | 33 | Standard | 70.0% |  |  |  | 10.0% | 90.0% |  |
|  | 20 | Hypofractionation | 10.0% |  |  |  |  | 100.0% |  |
| **Tumor site** | **N fractions** | **Fractionation schedule** | **%** | **Single field RT** | **2D-RT** | **3D-CRT** | **IMRT** | **Rotational IMRT** | **Stereotactic** |
| **Rectum** | 25 | Standard | 30.0% |  |  |  | 10.0% | 90.0% |  |
|  | 28 | Standard | 40.0% |  |  |  | 10.0% | 90.0% |  |
|  | 5 | Extreme hypofractionation | 30.0% |  |  |  | 10.0% | 90.0% |  |
| **Soft tissue** | 25 | Standard | 100.0% |  |  | 50.0% | 25.0% | 25.0% |  |
| **Stomach** | 25 | Standard | 100.0% |  |  | 25.0% | 25.0% | 50.0% |  |
| **Testis** | 15 | Hypofractionation | 96.0% |  |  | 25.0% | 25.0% | 50.0% |  |
|  | 20 | Hypofractionation | 4.0% |  |  | 25.0% | 25.0% | 50.0% |  |
| **Thyroid** | 25 | Standard | 26.0% |  |  |  | 10.0% | 90.0% |  |
|  | 28 | Standard | 30.0% |  |  |  | 10.0% | 90.0% |  |
|  | 29 | Standard | 44.0% |  |  |  | 10.0% | 90.0% |  |
| **Uterus** | 23 | Standard | 100.0% |  |  |  | 10.0% | 90.0% |  |
| **Vagina** | 25 | Standard | 100.0% |  |  |  | 10.0% | 90.0% |  |
| **Brain metastases** | 5 | Extreme hypofractionation | 40.0% |  |  |  |  | 20.0% |  |
|  | 10 | Extreme hypofractionation | 60.0% |  |  | 75.0% |  | 25.0% |  |
| **Bone metastases** | 1 | Extreme hypofractionation | 85.0% |  |  | 90.0% |  | 5.0% |  |
|  | 5 | Extreme hypofractionation | 10.0% |  |  | 90.0% |  | 10.0% |  |
|  | 10 | Extreme hypofractionation | 5.0% |  |  | 90.0% |  | 10.0% |  |
| **Other metastases** | 1 | Extreme hypofractionation | 20.0% |  |  | 80.0% | 5.0% | 15.0% |  |
|  | 5 | Extreme hypofractionation | 40.0% |  |  | 80.0% | 5.0% | 15.0% |  |
|  | 10 | Extreme hypofractionation | 40.0% |  |  | 80.0% | 5.0% | 15.0% |  |

2D-RT: 2D radiotherapy, 3D-CRT: 3-dimensional conformal RT, EBRT: external beam radiotherapy, IMRT: intensity-modulated RT, rotational IMRT: intensity-modulated rotational RT

**Supplementary Table 5.** Cost of EBRT-Core activities, RO-Support, and Beyond-EBRT, 2018 (€)

| **Resources** | **Total annual cost (€)** | **Percentage** |
| --- | --- | --- |
| **Total** | **42,198,713** | **100.0%** |
| **EBRT care pathway steps** | **29,143,904** | **69.0%** |
| Equipment | 15,915,380 | 37.7% |
| Personnel | 13,017,574 | 30.8% |
| Consumable | 210,950 | 0.5% |
| **RO Support** | **8,497,045** | **20.2%** |
| **Equipment** |  |  |
| R&V | 392,585 | 0.9% |
| Machine-specific dosimetry equipment | 495,000 | 1.2% |
| General IT equipment | 330,000 | 0.8% |
| General immobilization devices | 484,000 | 1.1% |
| General consumables | 797,500 | 1.9% |
| General spaces | 882,622 | 2.1% |
| **Personnel** |  |  |
| Administrative personnel | 1,338,440 | 3.2% |
| Quality manager | 273,642 | 0.6% |
| Social worker | 273,642 | 0.6% |
| **Activities** |  |  |
| Departmental management & team meetings | 1,272,843 | 3.0% |
| Quality assurance & quality management (excl. machine-related quality control & breakdowns) | 330,370 | 0.8% |
| Radiation safety & radiation protection | 490,674 | 1.2% |
| Technology & techniques implementation | 664,489 | 1.6% |
| Teaching (academic & on-the-job training) | 235,618 | 0.6% |
| Research | 235,618 | 0.6% |
| **Non-EBRT care** | **4,557,765** | **10.8%** |
| Multidisciplinary tumor boards | 869,077 | 2.1% |
| Bracyhtherapy & intraoperative therapy | 1,950,534 | 4.6% |
| Follow-up consultations | 1,738,154 | 4.1% |

EBRT: external beam radiotherapy, RO: radiation oncology, R&V: record and verification.

**Supplementary Table 6.** Average EBRT cost per treatment, technique, fractionation schedule, and activities, 2018 (€)

|  | **Fractionation schedule** | **Courses (n)** | **Average EBRT course cost (€)** | | | | | | | | | | | | | |
| --- | --- | --- | --- | --- | --- | --- | --- | --- | --- | --- | --- | --- | --- | --- | --- | --- |
|  |  |  | **Planning and related quality control** | |  | **Delivery and related quality control** | |  | **RO-support** | |  | **Non-EBRT** | |  | **Mean cost** | |
|  |  |  | **Euros (€)** | **%** |  | **Euros (€)** | **%** |  | **Euros (€)** | **%** |  | **Euros (€)** | **%** |  | **Euros (€)** | **%** |
| **Bladder** |  | **124** | **563** | **13.0** |  | **2366** | **54.5** |  | **918** | **21.2** |  | **493** | **11.4** |  | **4340** | **100.0** |
| Rotational IMRT | Standard | 124 | 563 | 13.0 |  | 2366 | 54.5 |  | 918 | 21.2 |  | 493 | 11.4 |  | 4340 | 100.0 |
| **Brain** |  | **353** | **656** | **17.5** |  | **1926** | **51.5** |  | **754** | **20.2** |  | **404** | **10.8** |  | **3740** | **100.0** |
| IMRT | Standard | 49 | 656 | 14.7 |  | 2470 | 55.4 |  | 865 | 19.4 |  | 464 | 10.4 |  | 4455 | 100.0 |
| IMRT | Hypofractionation | 21 | 656 | 24.6 |  | 1250 | 46.9 |  | 494 | 18.5 |  | 265 | 9.9 |  | 2665 | 100.0 |
| Rotational IMRT | Standard | 198 | 656 | 15.6 |  | 2209 | 52.7 |  | 865 | 20.6 |  | 464 | 11.1 |  | 4194 | 100.0 |
| Rotational IMRT | Hypofractionation | 85 | 656 | 25.9 |  | 1119 | 44.1 |  | 494 | 19.5 |  | 265 | 10.5 |  | 2534 | 100.0 |
| **Breast** |  | **3543** | **578** | **18.9** |  | **1545** | **50.6** |  | **607** | **19.9** |  | **325** | **10.7** |  | **3055** | **100.0** |
| 3D-CRT | Standard | 124 | 473 | 14.8 |  | 1481 | 46.2 |  | 815 | 25.4 |  | 437 | 13.6 |  | 3206 | 100.0 |
| 3D-CRT | Hypofractionation | 230 | 473 | 23.2 |  | 806 | 39.5 |  | 494 | 24.2 |  | 265 | 13.0 |  | 2039 | 100.0 |
| IMRT | Standard | 744 | 589 | 14.0 |  | 2361 | 56.2 |  | 815 | 19.4 |  | 437 | 10.4 |  | 4202 | 100.0 |
| IMRT | Hypofractionation | 1382 | 589 | 22.4 |  | 1280 | 48.7 |  | 494 | 18.8 |  | 265 | 10.1 |  | 2628 | 100.0 |
| Rotational IMRT | Standard | 372 | 589 | 14.9 |  | 2117 | 53.5 |  | 815 | 20.6 |  | 437 | 11.0 |  | 3959 | 100.0 |
| Rotational IMRT | Hypofractionation | 691 | 589 | 23.6 |  | 1148 | 46.0 |  | 494 | 19.8 |  | 265 | 10.6 |  | 2497 | 100.0 |
| **Cervix** |  | **162** | **560** | **14.8** |  | **2059** | **54.5** |  | **754** | **20.0** |  | **404** | **10.7** |  | **3777** | **100.0** |
| IMRT | Standard | 130 | 560 | 14.6 |  | 2104 | 55.1 |  | 754 | 19.7 |  | 404 | 10.6 |  | 3822 | 100.0 |
| Rotational IMRT | Standard | 32 | 560 | 15.5 |  | 1882 | 52.3 |  | 754 | 20.9 |  | 404 | 11.2 |  | 3600 | 100.0 |
| **Head and neck** |  | **840** | **715** | **16.4** |  | **2283** | **52.5** |  | **881** | **20.2** |  | **473** | **10.9** |  | **4351** | **100.0** |
| IMRT | Standard | 84 | 715 | 15.6 |  | 2523 | 54.9 |  | 881 | 19.2 |  | 473 | 10.3 |  | 4592 | 100.0 |
| Rotational IMRT | Standard | 756 | 715 | 16.5 |  | 2256 | 52.2 |  | 881 | 20.4 |  | 473 | 10.9 |  | 4325 | 100.0 |
| **Leukemia** |  | **31** | **444** | **5.9** |  | **625** | **365** |  | **420** | **24.5** |  | **225** | **13.1** |  | **1714** | **100.0** |
| 3D-CRT | Standard | 31 | 444 | 25.9 |  | 625 | 36.5 |  | 420 | 24.5 |  | 225 | 13.1 |  | 1714 | 100.0 |
|  |  |  |  |  |  |  |  |  |  |  |  |  |  |  |  |  |
| **Lung** |  | **1185** | **778** | **18.1** |  | **2308** | **53.7** |  | **787** | **18.3** |  | **422** | **9.8** |  | **4296** | **100.0** |
| IMRT | Standard | 563 | 773 | 17.1 |  | 2493 | 55.2 |  | 816 | 18.1 |  | 438 | 9.7 |  | 4519 | 100.0 |
| Rotational IMRT | Standard | 563 | 773 | 18.1 |  | 2249 | 52.6 |  | 816 | 19.1 |  | 438 | 10.2 |  | 4275 | 100.0 |
| Stereotactic techniques |  | 59 | 882 | 37.1 |  | 1119 | 47.1 |  | 245 | 10.3 |  | 131 | 5.5 |  | 2377 | 100.0 |
| **Lymphoma** |  | **318** | **595** | **26.1** |  | **1020** | **44.8** |  | **432** | **19.0** |  | **232** | **10.2** |  | **2279** | **100.0** |
| IMRT | Standard | 111 | 595 | 22.8 |  | 1260 | 48.2 |  | 494 | 18.9 |  | 265 | 10.1 |  | 2615 | 100.0 |
| IMRT | Extreme hypofractionation | 111 | 595 | 29.6 |  | 846 | 42.1 |  | 370 | 18.4 |  | 198 | 9.9 |  | 2009 | 100.0 |
| Rotational IMRT | Standard | 48 | 595 | 24.0 |  | 1129 | 45.4 |  | 494 | 19.9 |  | 265 | 10.7 |  | 2483 | 100.0 |
| Rotational IMRT | Extreme hypofractionation | 48 | 595 | 31.0 |  | 758 | 39.5 |  | 370 | 19.2 |  | 198 | 10.3 |  | 1922 | 100.0 |
| **Melanoma** |  | **394** | **511** | **15.6** |  | **1621** | **49.5** |  | **744** | **22.7** |  | **399** | **12.2** |  | **3275** | **100.0** |
| 3D-CRT | Standard | 99 | 453 | 13.6 |  | 1534 | 46.2 |  | 869 | 26.2 |  | 466 | 14.0 |  | 3321 | 100.0 |
| 3D-CRT | Hypofractionation | 99 | 453 | 18.6 |  | 1029 | 42.3 |  | 619 | 25.4 |  | 332 | 13.6 |  | 2433 | 100.0 |
| IMRT | Standard | 49 | 569 | 13.0 |  | 2482 | 56.6 |  | 869 | 19.8 |  | 466 | 10.6 |  | 4385 | 100.0 |
| IMRT | Hypofractionation | 49 | 569 | 17.9 |  | 1660 | 52.2 |  | 619 | 19.5 |  | 332 | 10.4 |  | 3180 | 100.0 |
| Rotational IMRT | Standard | 49 | 569 | 13.8 |  | 2219 | 53.8 |  | 869 | 21.1 |  | 466 | 11.3 |  | 4122 | 100.0 |
| Rotational IMRT | Hypofractionation | 49 | 569 | 18.9 |  | 1486 | 49.4 |  | 619 | 20.6 |  | 332 | 11.0 |  | 3005 | 100.0 |
| **Myeloma** |  | **56** | **582** | **18.5** |  | **1608** | **51.2** |  | **619** | **19.7** |  | **332** | **10.6** |  | **3141** | **100.0** |
| IMRT | Standard | 39 | 582 | 18.2 |  | 1660 | 52.0 |  | 619 | 19.4 |  | 332 | 10.4 |  | 3194 | 100.0 |
| Rotational IMRT | Standard | 17 | 582 | 19.3 |  | 1486 | 49.2 |  | 619 | 20.5 |  | 332 | 11.0 |  | 3019 | 100.0 |
| **Esophagus** |  | **190** | **640** | **15.2** |  | **2303** | **54.8** |  | **819** | **19.5** |  | **439** | **10.5** |  | **4201** | **100.0** |
| IMRT | Standard | 133 | 640 | 15.0 |  | 2376 | 55.6 |  | 819 | 19.2 |  | 439 | 10.3 |  | 4274 | 100.0 |
| Rotational IMRT | Standard | 57 | 640 | 15.9 |  | 2132 | 52.9 |  | 819 | 20.3 |  | 439 | 10.9 |  | 4029 | 100.0 |
|  |  |  |  |  |  |  |  |  |  |  |  |  |  |  |  |  |
|  |  |  |  |  |  |  |  |  |  |  |  |  |  |  |  |  |
| **Pancreas** |  | **130** | **822** | **17.7** |  | **2564** | **55.2** |  | **819** | **17.6** |  | **439** | **9.5** |  | **4645** | **100.0** |
| IMRT | Standard | 65 | 822 | 17.3 |  | 2687 | 56.4 |  | 819 | 17.2 |  | 439 | 9.2 |  | 4767 | 100.0 |
| Rotational IMRT | Standard | 65 | 822 | 18.2 |  | 2442 | 54.0 |  | 819 | 18.1 |  | 439 | 9.7 |  | 4522 | 100.0 |
| **Prostate** |  | **1765** | **563** | **13.4** |  | **2291** | **54.3** |  | **886** | **21.0** |  | **475** | **11.3** |  | **4215** | **100.0** |
| IMRT | Standard | 124 | 563 | 11.9 |  | 2728 | 57.5 |  | 943 | 19.9 |  | 506 | 10.7 |  | 4740 | 100.0 |
| Rotational IMRT | Standard | 1465 | 563 | 13.0 |  | 2351 | 54.5 |  | 913 | 21.2 |  | 490 | 11.3 |  | 4317 | 100.0 |
| Rotational IMRT | Hypofractionation | 177 | 563 | 18.8 |  | 1486 | 49.5 |  | 619 | 20.6 |  | 332 | 11.1 |  | 3000 | 100.0 |
| **Rectum** |  | **803** | **563** | **18.3** |  | **1553** | **50.5** |  | **624** | **20.3** |  | **335** | **10.9** |  | **3075** | **100.0** |
| IMRT | Standard | 169 | 563 | 14.1 |  | 2212 | 55.5 |  | 787 | 19.7 |  | 422 | 10.6 |  | 3983 | 100.0 |
| IMRT | Extreme hypofractionation | 72 | 563 | 41.2 |  | 429 | 31.3 |  | 245 | 17.9 |  | 131 | 9.6 |  | 1368 | 100.0 |
| Rotational IMRT | Standard | 393 | 563 | 15.0 |  | 1978 | 52.8 |  | 787 | 21.0 |  | 422 | 11.3 |  | 3750 | 100.0 |
| Rotational IMRT | Extreme hypofractionation | 169 | 563 | 42.5 |  | 385 | 29.1 |  | 245 | 18.5 |  | 131 | 9.9 |  | 1324 | 100.0 |
| **Soft tissue** |  | **182** | **547** | **16.5** |  | **1621** | **49.0** |  | **744** | **22.5** |  | **399** | **12.1** |  | **3311** | **100.0** |
| 3D-CRT | Standard | 91 | 489 | 16.8 |  | 1281 | 44.0 |  | 744 | 25.5 |  | 399 | 13.7 |  | 2913 | 100.0 |
| IMRT | Standard | 46 | 604 | 15.8 |  | 2071 | 54.2 |  | 744 | 19.5 |  | 399 | 10.4 |  | 3818 | 100.0 |
| Rotational IMRT | Standard | 46 | 604 | 16.8 |  | 1852 | 51.5 |  | 744 | 20.7 |  | 399 | 11.1 |  | 3600 | 100.0 |
| **Stomach** |  | **75** | **505** | **15.4** |  | **1621** | **49.6** |  | **744** | **22.8** |  | **399** | **12.2** |  | **3269** | **100.0** |
| 3D-CRT | Standard | 38 | 447 | 15.6 |  | 1281 | 44.6 |  | 744 | 25.9 |  | 399 | 13.9 |  | 2871 | 100.0 |
| IMRT | Standard | 19 | 563 | 14.9 |  | 2071 | 54.8 |  | 744 | 19.7 |  | 399 | 10.6 |  | 3777 | 100.0 |
| Rotational IMRT | Standard | 19 | 563 | 15.8 |  | 1852 | 52.1 |  | 744 | 20.9 |  | 399 | 11.2 |  | 3558 | 100.0 |
| **Testis** |  | **13** | **502** | **22.2** |  | **993** | **43.9** |  | **499** | **22.1** |  | **268** | **11.8** |  | **2262** | **100.0** |
| 3D-CRT | Standard | 7 | 444 | 22.2 |  | 787 | 39.4 |  | 499 | 25.0 |  | 268 | 13.4 |  | 1998 | 100.0 |
| IMRT | Standard | 3 | 560 | 21.6 |  | 1266 | 48.8 |  | 499 | 19.3 |  | 268 | 10.3 |  | 2593 | 100.0 |
| Rotational IMRT | Standard | 3 | 560 | 22.7 |  | 1133 | 46.1 |  | 499 | 20.3 |  | 268 | 10.9 |  | 2460 | 100.0 |
| **Thyroid** |  | **13** | **653** | **16.1** |  | **2169** | **53.3** |  | **810** | **19.9** |  | **435** | **10.7** |  | **4066** | **100.0** |
| IMRT | Standard | 6.5 | 653 | 15.6 |  | 2289 | 54.7 |  | 810 | 19.4 |  | 435 | 10.4 |  | 4187 | 100.0 |
| Rotational IMRT | Standard | 6.5 | 653 | 16.5 |  | 2048 | 51.9 |  | 810 | 20.5 |  | 435 | 11.0 |  | 3945 | 100.0 |
| **Uterus** |  | **230** | **563** | **16.4** |  | **1806** | **52.6** |  | **694** | **20.2** |  | **372** | **10.8** |  | **3435** | **100.0** |
| IMRT | Standard | 115 | 563 | 15.9 |  | 1907 | 53.9 |  | 694 | 19.6 |  | 372 | 10.5 |  | 3536 | 100.0 |
| Rotational IMRT | Standard | 115 | 563 | 16.9 |  | 1706 | 51.1 |  | 694 | 20.8 |  | 372 | 11.2 |  | 3335 | 100.0 |
| **Vagina** |  | **77** | **563** | **15.4** |  | **1962** | **53.5** |  | **744** | **20.3** |  | **399** | **10.9** |  | **3667** | **100.0** |
| IMRT | Standard | 39 | 563 | 14.9 |  | 2071 | 54.8 |  | 744 | 19.7 |  | 399 | 10.6 |  | 3777 | 100.0 |
| Rotational IMRT | Standard | 39 | 563 | 15.8 |  | 1852 | 52.1 |  | 744 | 20.9 |  | 399 | 11.2 |  | 3558 | 100.0 |
| **Bone metastases** |  | **914** | **490** | **52.2** |  | **173** | **18.5** |  | **179** | **19.1** |  | **96** | **10.2** |  | **938** | **100.0** |
| 3D-CRT | Extreme hypofractionation | 626 | 453 | 53.2 |  | 130 | 15.3 |  | 175 | 20.5 |  | 94 | 11.0 |  | 851 | 100.0 |
| IMRT | Extreme hypofractionation | 197 | 569 | 50.8 |  | 255 | 22.8 |  | 192 | 17.2 |  | 103 | 9.2 |  | 1119 | 100.0 |
| Rotational IMRT | Extreme hypofractionation | 85 | 569 | 58.9 |  | 145 | 15.0 |  | 163 | 16.9 |  | 88 | 9.1 |  | 965 | 100.0 |
| Stereotactic techniques |  | 7 | 672 | 19.8 |  | 2154 | 63.5 |  | 370 | 10.9 |  | 198 | 5.8 |  | 3393 | 100.0 |
| **Brain metastases** |  | **573** | **615** | **35.1** |  | **646** | **36.9** |  | **320** | **18.2** |  | **172** | **9.8** |  | **1753** | **100.0** |
| 3D-CRT | Extreme hypofractionation | 332 | 534 | 33.4 |  | 507 | 31.8 |  | 361 | 22.6 |  | 194 | 12.1 |  | 1595 | 100.0 |
| Rotational IMRT | Extreme hypofractionation | 103 | 649 | 40.7 |  | 507 | 31.8 |  | 287 | 17.9 |  | 154 | 9.6 |  | 1597 | 100.0 |
| Stereotactic techniques |  | 138 | 788 | 35.0 |  | 1086 | 48.3 |  | 245 | 10.9 |  | 131 | 5.8 |  | 2250 | 100.0 |
| **Other metastases** |  | **2167** | **476** | **37.6** |  | **366** | **29.0** |  | **275** | **21.7** |  | **147** | **11.7** |  | **1264** | **100.0** |
| 3D-CRT | Extreme hypofractionation | 1734 | 453 | 37.5 |  | 333 | 27.5 |  | 275 | 22.8 |  | 147 | 12.2 |  | 1208 | 100.0 |
| IMRT | Extreme hypofractionation | 217 | 569 | 37.5 |  | 527 | 34.7 |  | 275 | 18.1 |  | 147 | 9.7 |  | 1518 | 100.0 |
| Rotational IMRT | Extreme hypofractionation | 217 | 569 | 38.8 |  | 473 | 32.3 |  | 275 | 18.8 |  | 147 | 10.1 |  | 1464 | 100.0 |

3D-CRT: 3-dimensional conformal RT, IMRT: intensity modulated RT, rotational IMRT: intensity-modulated rotational RT

**Supplementary Table 7.** Average EBRT cost per treatment, technique, fractionation schedule, and activities, 2023 (€)

|  | **Fractionation schedule** | **Courses (n)** | **Average EBRT course cost (€)** | | | | | | | | | | | | | | |
| --- | --- | --- | --- | --- | --- | --- | --- | --- | --- | --- | --- | --- | --- | --- | --- | --- | --- |
|  |  |  | **Planning & related quality control** | |  | **Delivery & related quality control** | |  | **RO-support** | |  | **Non-EBRT** | |  | **Mean cost** | | |
|  |  |  | **Euros (€)** | **%** |  | **Euros (€)** | **%** |  | **Euros (€)** | **%** |  | **Euros (€)** | **%** |  | **Euros (€)** | | **%** |
| **Bladder** |  | **124** | **581** | **12.8** |  | **2464** | **54.5** |  | **963** | **21.3** |  | **516** | **11.4** |  | **4524** | **100.0** | |
| Rotational IMRT | Standard | 124 | 581 | 12.8 |  | 2464 | 54.5 |  | 963 | 21.3 |  | 516 | 11.4 |  | 4524 | 100.0 | |
| **Brain** |  | **353** | **667** | **17.4** |  | **1960** | **51.0** |  | **789** | **20.6** |  | **423** | **11.0** |  | **3839** | **100.0** | |
| IMRT | Standard | 0 | 0 | 0.0 |  | 0 | 0.0 |  | 0 | 0.0 |  | 0 | 0.0 |  | 0 | 0.0 | |
| IMRT | Hypofractionation | 0 | 0 | 0.0 |  | 0 | 0.0 |  | 0 | 0.0 |  | 0 | 0.0 |  | 0 | 0.0 | |
| Rotational IMRT | Standard | 247 | 667 | 15.3 |  | 2300 | 52.8 |  | 906 | 20.8 |  | 486 | 11.1 |  | 4360 | 100.0 | |
| Rotational IMRT | Hypofractionation | 106 | 667 | 25.4 |  | 1165 | 44.4 |  | 515 | 19.6 |  | 276 | 10.5 |  | 2624 | 100.0 | |
| **Breast** |  | **3543** | **619** | **19.4** |  | **1636** | **51.3** |  | **610** | **19.1** |  | **327** | **10.3** |  | **3192** | **100.0** | |
| 3D-CRT | Standard | 213 | 526 | 14.5 |  | 1829 | 50.4 |  | 831 | 22.9 |  | 446 | 12.3 |  | 3632 | 100.0 | |
| 3D-CRT | Hypofractionation | 496 | 526 | 22.5 |  | 1024 | 43.7 |  | 515 | 22.0 |  | 276 | 11.8 |  | 2342 | 100.0 | |
| IMRT | Standard | 531 | 642 | 14.1 |  | 2640 | 57.9 |  | 831 | 18.2 |  | 446 | 9.8 |  | 4559 | 100.0 | |
| IMRT | Hypofractionation | 1240 | 642 | 22.1 |  | 1475 | 50.7 |  | 515 | 17.7 |  | 276 | 9.5 |  | 2908 | 100.0 | |
| Rotational IMRT | Standard | 319 | 642 | 15.3 |  | 2268 | 54.2 |  | 831 | 19.8 |  | 446 | 10.6 |  | 4187 | 100.0 | |
| Rotational IMRT | Hypofractionation | 744 | 642 | 23.8 |  | 1268 | 46.9 |  | 515 | 19.1 |  | 276 | 10.2 |  | 2702 | 100.0 | |
| **Cervix** |  | **162** | **597** | **15.7** |  | **1995** | **52.4** |  | **789** | **20.7** |  | **423** | **11.1** |  | **3804** | **100.0** | |
| IMRT | Standard | 16 | 597 | 14.5 |  | 2310 | 56.1 |  | 789 | 19.2 |  | 423 | 10.3 |  | 4119 | 100.0 | |
| Rotational IMRT | Standard | 146 | 597 | 15.8 |  | 1960 | 52.0 |  | 789 | 20.9 |  | 423 | 11.2 |  | 3769 | 100.0 | |
| **Head and neck** |  | **840** | **699** | **15.5** |  | **2392** | **53.0** |  | **923** | **20.5** |  | **495** | **11.0** |  | **4509** | **100.0** | |
| IMRT | Standard | 84 | 699 | 14.3 |  | 2770 | 56.7 |  | 923 | 18.9 |  | 495 | 10.1 |  | 4887 | 100.0 | |
| Rotational IMRT | Standard | 756 | 699 | 15.7 |  | 2350 | 52.6 |  | 923 | 20.7 |  | 495 | 11.1 |  | 4467 | 100.0 | |
| **Leukemia** |  | **31** | **433** | **23.5** |  | **741** | **40.2** |  | **436** | **23.7** |  | **234** | **12.7** |  | **1843** | **100.0** | |
| 3D-CRT | Standard | 31 | 433 | 23.5 |  | 741 | 40.2 |  | 436 | 23.7 |  | 234 | 12.7 |  | 1843 | 100.0 | |
|  |  |  |  |  |  |  |  |  |  |  |  |  |  |  |  |  | |
|  |  |  |  |  |  |  |  |  |  |  |  |  |  |  |  |  | |
| **Lung** |  | **1185** | **777** | **21.8** |  | **1827** | **51.4** |  | **620** | **17.4** |  | **333** | **9.4** |  | **3558** | **100.0** | |
| 3D-CRT | Standard | 356 | 651 | 16.5 |  | 1957 | 49.7 |  | 866 | 22.0 |  | 465 | 11.8 |  | 3939 | 100.0 | |
| IMRT | Standard | 142 | 767 | 15.6 |  | 2808 | 57.2 |  | 866 | 17.7 |  | 465 | 9.5 |  | 4906 | 100.0 | |
| Rotational IMRT | Standard | 213 | 767 | 17.0 |  | 2418 | 53.5 |  | 866 | 19.2 |  | 465 | 10.3 |  | 4516 | 100.0 | |
| Stereotactic techniques |  | 474 | 879 | 36.1 |  | 1170 | 48.0 |  | 252 | 10.3 |  | 135 | 5.5 |  | 2437 | 100.0 | |
| **Lymphoma** |  | **318** | **591** | **26.4** |  | **958** | **42.8** |  | **449** | **20.1** |  | **241** | **10.8** |  | **2239** | **100.0** | |
| 3D-CRT | Standard | 40 | 504 | 22.6 |  | 931 | 41.8 |  | 515 | 23.1 |  | 276 | 12.4 |  | 2227 | 100.0 | |
| 3D-CRT | Extreme hypofractionation | 40 | 504 | 29.3 |  | 627 | 36.4 |  | 383 | 22.3 |  | 206 | 12.0 |  | 1721 | 100.0 | |
| IMRT | Standard | 24 | 620 | 22.2 |  | 1382 | 49.5 |  | 515 | 18.4 |  | 276 | 9.9 |  | 2794 | 100.0 | |
| IMRT | Extreme hypofractionation | 24 | 620 | 29.0 |  | 928 | 43.4 |  | 383 | 17.9 |  | 206 | 9.6 |  | 2137 | 100.0 | |
| Rotational IMRT | Standard | 95 | 620 | 24.0 |  | 1175 | 45.4 |  | 515 | 19.9 |  | 276 | 10.7 |  | 2587 | 100.0 | |
| Rotational IMRT | Extreme hypofractionation | 95 | 620 | 31.0 |  | 790 | 39.5 |  | 383 | 19.2 |  | 206 | 10.3 |  | 1999 | 100.0 | |
| **Melanoma** |  | **394** | **499** | **14.6** |  | **1726** | **50.5** |  | **778** | **22.8** |  | **418** | **12.2** |  | **3421** | **100.0** | |
| 3D-CRT | Standard | 99 | 441 | 12.0 |  | 1823 | 49.8 |  | 910 | 24.8 |  | 488 | 13.3 |  | 3663 | 100.0 | |
| 3D-CRT | Hypofractionation | 99 | 441 | 16.6 |  | 1222 | 46.0 |  | 647 | 24.3 |  | 347 | 13.1 |  | 2657 | 100.0 | |
| IMRT | Standard | 0 | 0 | 0.0 |  | 0 | 0.0 |  | 0 | 0.0 |  | 0 | 0.0 |  | 0 | 0.0 | |
| IMRT | Hypofractionation | 0 | 0 | 0.0 |  | 0 | 0.0 |  | 0 | 0.0 |  | 0 | 0.0 |  | 0 | 0.0 | |
| Rotational IMRT | Standard | 99 | 557 | 13.1 |  | 2311 | 54.2 |  | 910 | 21.3 |  | 488 | 11.4 |  | 4267 | 100.0 | |
| Rotational IMRT | Hypofractionation | 99 | 557 | 18.0 |  | 1547 | 49.9 |  | 647 | 20.9 |  | 347 | 11.2 |  | 3098 | 100.0 | |
| **Myeloma** |  | **56** | **454** | **17.0** |  | **1222** | **45.8** |  | **647** | **24.2** |  | **347** | **13.0** |  | **2670** | **100.0** | |
| 3D-CRT | Standard | 56 | 454 | 17.0 |  | 1222 | 45.8 |  | 647 | 24.2 |  | 347 | 13.0 |  | 2670 | 100.0 | |
| **Esophagus** |  | **190** | **686** | **16.2** |  | **2220** | **52.6** |  | **857** | **20.3** |  | **460** | **10.9** |  | **4223** | **100.0** | |
| IMRT | Standard | 0 | 0 | 0.0 |  | 0 | 0.0 |  | 0 | 0.0 |  | 0 | 0.0 |  | 0 | 0.0 | |
| Rotational IMRT | Standard | 190 | 686 | 16.2 |  | 2220 | 52.6 |  | 857 | 20.3 |  | 460 | 10.9 |  | 4223 | 100.0 | |
| **Pancreas** |  | **130** | **837** | **23.9** |  | **1820** | **51.9** |  | **555** | **15.8** |  | **298** | **8.5** |  | **3509** | **100.0** | |
| 3D-CRT | Standard | 23 | 692 | 16.9 |  | 2089 | 51.0 |  | 857 | 20.9 |  | 460 | 11.2 |  | 4098 | 100.0 | |
| IMRT | Standard | 10 | 808 | 16.0 |  | 2930 | 58.0 |  | 857 | 17.0 |  | 460 | 9.1 |  | 5055 | 100.0 | |
| Rotational IMRT | Standard | 33 | 808 | 17.3 |  | 2544 | 54.5 |  | 857 | 18.4 |  | 460 | 9.8 |  | 4670 | 100.0 | |
| Stereotactic techniques |  | 65 | 906 | 36.4 |  | 1198 | 48.1 |  | 252 | 10.1 |  | 135 | 5.4 |  | 2491 | 100.0 | |
| **Prostate** |  | **1765** | **577** | **13.1** |  | **2418** | **54.7** |  | **929** | **21.0** |  | **498** | **11.3** |  | **4422** | **100.0** | |
| IMRT | Standard | 124 | 577 | 11.3 |  | 3018 | 59.0 |  | 989 | 19.3 |  | 531 | 10.4 |  | 5115 | 100.0 | |
| Rotational IMRT | Standard | 1465 | 577 | 12.8 |  | 2470 | 54.7 |  | 957 | 21.2 |  | 514 | 11.4 |  | 4518 | 100.0 | |
| Rotational IMRT | Hypofractionation | 177 | 577 | 18.4 |  | 1561 | 49.8 |  | 647 | 20.7 |  | 347 | 11.1 |  | 3132 | 100.0 | |
| **Rectum** |  | **803** | **597** | **18.7** |  | **1590** | **49.9** |  | **652** | **20.4** |  | **350** | **11.0** |  | **3189** | **100.0** | |
| IMRT | Standard | 56 | 597 | 13.9 |  | 2428 | 56.6 |  | 824 | 19.2 |  | 442 | 10.3 |  | 4290 | 100.0 | |
| IMRT | Extreme hypofractionation | 24 | 597 | 41.1 |  | 470 | 32.3 |  | 252 | 17.3 |  | 135 | 9.3 |  | 1453 | 100.0 | |
| Rotational IMRT | Standard | 506 | 597 | 15.2 |  | 2060 | 52.5 |  | 824 | 21.0 |  | 442 | 11.3 |  | 3922 | 100.0 | |
| Rotational IMRT | Extreme hypofractionation | 217 | 597 | 43.1 |  | 401 | 29.0 |  | 252 | 18.2 |  | 135 | 9.8 |  | 1385 | 100.0 | |
| **Soft tissue** |  | **182** | **534** | **15.1** |  | **1812** | **51.2** |  | **778** | **22.0** |  | **418** | **11.8** |  | **3542** | **100.0** | |
| 3D-CRT | Standard | 91 | 476 | 14.9 |  | 1523 | 47.7 |  | 778 | 24.4 |  | 418 | 13.1 |  | 3194 | 100.0 | |
| IMRT | Standard | 46 | 592 | 14.6 |  | 2274 | 56.0 |  | 778 | 19.2 |  | 418 | 10.3 |  | 4061 | 100.0 | |
| Rotational IMRT | Standard | 46 | 592 | 15.9 |  | 1929 | 51.9 |  | 778 | 20.9 |  | 418 | 11.2 |  | 3717 | 100.0 | |
| **Stomach** |  | **75** | **584** | **15.8** |  | **1914** | **51.8** |  | **778** | **21.1** |  | **418** | **11.3** |  | **3693** | **100.0** | |
| 3D-CRT | Standard | 19 | 497 | 15.4 |  | 1523 | 47.4 |  | 778 | 24.2 |  | 418 | 13.0 |  | 3215 | 100.0 | |
| IMRT | Standard | 19 | 613 | 15.0 |  | 2274 | 55.7 |  | 778 | 19.1 |  | 418 | 10.2 |  | 4082 | 100.0 | |
| Rotational IMRT | Standard | 38 | 613 | 16.4 |  | 1929 | 51.6 |  | 778 | 20.8 |  | 418 | 11.2 |  | 3738 | 100.0 | |
|  |  |  |  |  |  |  |  |  |  |  |  |  |  |  |  |  | |
|  |  |  |  |  |  |  |  |  |  |  |  |  |  |  |  |  | |
| **Testis** |  | **13** | **520** | **20.9** |  | **1171** | **47.0** |  | **520** | **20.9** |  | **279** | **11.2** |  | **2490** | **100.0** | |
| 3D-CRT | Standard | 3 | 433 | 20.0 |  | 933 | 43.1 |  | 520 | 24.0 |  | 279 | 12.9 |  | 2165 | 100.0 | |
| IMRT | Standard | 3 | 549 | 20.0 |  | 1390 | 50.8 |  | 520 | 19.0 |  | 279 | 10.2 |  | 2738 | 100.0 | |
| Rotational IMRT | Standard | 7 | 549 | 21.7 |  | 1180 | 46.7 |  | 520 | 20.6 |  | 279 | 11.0 |  | 2528 | 100.0 | |
| **Thyroid** |  | **13** | **699** | **16.8** |  | **2171** | **52.0** |  | **848** | **20.3** |  | **455** | **10.9** |  | **4173** | **100.0** | |
| IMRT | Standard | 1 | 699 | 15.5 |  | 2513 | 55.7 |  | 848 | 18.8 |  | 455 | 10.1 |  | 4516 | 100.0 | |
| Rotational IMRT | Standard | 12 | 699 | 16.9 |  | 2133 | 51.6 |  | 848 | 20.5 |  | 455 | 11.0 |  | 4135 | 100.0 | |
| **Uterus** |  | **230** | **587** | **16.7** |  | **1808** | **51.5** |  | **726** | **20.7** |  | **389** | **11.1** |  | **3510** | **100.0** | |
| IMRT | Standard | 23 | 587 | 15.5 |  | 2093 | 55.2 |  | 726 | 19.1 |  | 389 | 10.3 |  | 3795 | 100.0 | |
| Rotational IMRT | Standard | 207 | 587 | 16.9 |  | 1776 | 51.1 |  | 726 | 20.9 |  | 389 | 11.2 |  | 3479 | 100.0 | |
| **Vagina** |  | **77** | **613** | **16.2** |  | **1964** | **52.1** |  | **778** | **20.6** |  | **418** | **11.1** |  | **3772** | **100.0** | |
| IMRT | Standard | 8 | 613 | 15.0 |  | 2274 | 55.7 |  | 778 | 19.1 |  | 418 | 10.2 |  | 4082 | 100.0 | |
| Rotational IMRT | Standard | 69 | 613 | 16.4 |  | 1929 | 51.6 |  | 778 | 20.8 |  | 418 | 11.2 |  | 3738 | 100.0 | |
| **Bone metastases** |  | **914** | **457** | **53.4** |  | **139** | **16.3** |  | **169** | **19.7** |  | **91** | **10.6** |  | **856** | **100.0** | |
| 3D-CRT | Extreme hypofractionation | 823 | 441 | 53.1 |  | 130 | 15.6 |  | 169 | 20.3 |  | 91 | 10.9 |  | 831 | 100.0 | |
| IMRT | Extreme hypofractionation | 0 | 0 | 0.0 |  | 0 | 0.0 |  | 0 | 0.0 |  | 0 | 0.0 |  | 0 | 0.0 | |
| Rotational IMRT | Extreme hypofractionation | 53 | 557 | 53.1 |  | 208 | 19.8 |  | 185 | 17.7 |  | 99 | 9.5 |  | 1050 | 100.0 | |
| Stereotactic techniques |  | 39 | 655 | 58.4 |  | 241 | 21.5 |  | 147 | 13.1 |  | 79 | 7.0 |  | 1121 | 100.0 | |
|  |  |  |  |  |  |  |  |  |  |  |  |  |  |  |  |  | |
|  |  |  |  |  |  |  |  |  |  |  |  |  |  |  |  |  | |
|  |  |  |  |  |  |  |  |  |  |  |  |  |  |  |  |  | |
|  |  |  |  |  |  |  |  |  |  |  |  |  |  |  |  |  | |
|  |  |  |  |  |  |  |  |  |  |  |  |  |  |  |  |  | |
|  |  |  |  |  |  |  |  |  |  |  |  |  |  |  |  |  | |
| **Brain metastases** |  | **573** | **644** | **33.2** |  | **790** | **40.7** |  | **331** | **17.0** |  | **177** | **9.1** |  | **1942** | **100.0** | |
| 3D-CRT | Extreme hypofractionation | 258 | 538 | 30.8 |  | 620 | 35.5 |  | 383 | 21.9 |  | 206 | 11.8 |  | 1748 | 100.0 | |
| Rotational IMRT | Extreme hypofractionation | 132 | 654 | 35.9 |  | 650 | 35.7 |  | 338 | 18.5 |  | 181 | 9.9 |  | 1823 | 100.0 | |
| Stereotactic techniques |  | 183 | 785 | 34.1 |  | 1129 | 49.1 |  | 252 | 10.9 |  | 135 | 5.9 |  | 2301 | 100.0 | |
| **Other metastases** |  | **2167** | **464** | **35.3** |  | **416** | **31.6** |  | **283** | **21.5** |  | **152** | **11.6** |  | **1316** | **100.0** | |
| 3D-CRT | Extreme hypofractionation | 1734 | 441 | 34.8 |  | 392 | 30.9 |  | 283 | 22.3 |  | 152 | 12.0 |  | 1268 | 100.0 | |
| IMRT | Extreme hypofractionation | 108 | 557 | 35.5 |  | 578 | 36.8 |  | 283 | 18.0 |  | 152 | 9.7 |  | 1571 | 100.0 | |
| Rotational IMRT | Extreme hypofractionation | 325 | 557 | 37.5 |  | 493 | 33.2 |  | 283 | 19.1 |  | 152 | 10.2 |  | 1485 | 100.0 | |

3D-CRT: 3 Dimensional conformal RT, EBRT: external beam radiotherapy, IMRT: Intensity modulated RT, rotational IMRT: intensity-modulated rotational RT

**Supplementary Table 8.** Comparison of mean cost per EBRT treatment, technique, and fractionation schedule, by techniques and fractionation schedules, 2018 and 2023

|  | **Fractionation schedule** | **Mean EBRT treatment cost (€)** | | | | | | | |
| --- | --- | --- | --- | --- | --- | --- | --- | --- | --- |
|  |  | **% techniques and fractionation schedules 2018** | | |  | **% techniques and fractionation schedules 2023** | | | **Variation 2018-23 (%)** |
|  |  | **Courses (n)** | **%** | **Mean cost (€)** |  | **Courses (n)** | **%** | **Mean cost (€)** |  |
| **Bladder** |  | **124** | **100.0** | **4340** |  | **124** | **100.0** | **4524** | **4.2** |
| Rotational IMRT | Standard | 124 | 100.0 | 4340 |  | 124 | 100.0 | 4524 | 4.2 |
| **Brain** |  | **353** | **100.0** | **3740** |  | **353** | **100.0** | **3839** | **2.6** |
| IMRT | Standard | 49 | 14.0 | 4455 |  | - |  |  | −100.0 |
| IMRT | Hypofractionation | 21 | 6.0 | 2665 |  | - |  |  | −100.0 |
| Rotational IMRT | Standard | 198 | 56.0 | 4194 |  | 247 | 70.0 | 4360 | 4.0 |
| Rotational IMRT | Hypofractionation | 85 | 24.0 | 2534 |  | 106 | 30.0 | 2624 | 3.5 |
| **Breast** |  | **3543** | **100.0** | **3055** |  | **3543** | **100.0** | **3192** | **4.5** |
| 3D-CRT | Standard | 124 | 3.5 | 3206 |  | 213 | 6.0 | 3632 | 13.3 |
| 3D-CRT | Hypofractionation | 230 | 6.5 | 2039 |  | 496 | 14.0 | 2342 | 14.8 |
| IMRT | Standard | 744 | 21.0 | 4202 |  | 531 | 15.0 | 4559 | 8.5 |
| IMRT | Hypofractionation | 1,382 | 39.0 | 2628 |  | 1240 | 35.0 | 2908 | 10.7 |
| Rotational IMRT | Standard | 372 | 10.5 | 3959 |  | 319 | 9.0 | 4187 | 5.8 |
| Rotational IMRT | Hypofractionation | 691 | 19.5 | 2497 |  | 744 | 21.0 | 2702 | 8.2 |
| **Cervix** |  | **162** | **100.0** | **3777** |  | **162** | **100.0** | **3804** | **0.7** |
| IMRT | Standard | 130 | 80.0 | 3822 |  | 16 | 10.0 | 4119 | 7.8 |
| Rotational IMRT | Standard | 32 | 20.0 | 3600 |  | 146 | 90.0 | 3769 | 4.7 |
| **Head and neck** |  | **840** | **100.0** | **4351** |  | **840** | **100.0** | **4509** | **3.6** |
| IMRT | Standard | 84 | 10.0 | 4592 |  | 84 | 10.0 | 4887 | 6.4 |
| Rotational IMRT | Standard | 756 | 90.0 | 4325 |  | 756 | 90.0 | 4467 | 3.3 |
| **Leukemia** |  | **31** | **100.0** | **1714** |  | **31** | **100.0** | **1843** | **7.6** |
| 3D-CRT | Standard | 31 | 100.0 | 1714 |  | 31 | 100.0 | 1843 | 7.6 |
| **Lung** |  | **1185** | **100.0** | **4296** |  | **1185** | **100.0** | **3558** | −**17.2** |
| 3D-CRT | Standard | - |  |  |  | 356 | 30.0 | 3939 |  |
| IMRT | Standard | 563 | 47.5 | 4519 |  | 142 | 12.0 | 4906 | 8.6 |
| Rotational IMRT | Standard | 563 | 47.5 | 4275 |  | 213 | 18.0 | 4516 | 5.6 |
| Stereotactic techniques |  | 59 | 5.0 | 2377 |  | 474 | 40.0 | 2437 | 2.5 |
| **Lymphoma** |  | **318** | **100.0** | **2279** |  | **318** | **100.0** | **2239** | −**1.7** |
| 3D-CRT | Standard | - |  |  |  | 40 | 12.5 | 2227 |  |
| 3D-CRT | Extreme hypofractionation | - |  |  |  | 40 | 12.5 | 1721 |  |
| IMRT | Standard | 111 | 35.0 | 2615 |  | 24 | 7.5 | 2794 | 6.9 |
| IMRT | Extreme hypofractionation | 111 | 35.0 | 2009 |  | 24 | 7.5 | 2137 | 6.4 |
| Rotational IMRT | Standard | 48 | 15.0 | 2483 |  | 95 | 30.0 | 2587 | 4.2 |
| Rotational IMRT | Extreme hypofractionation | 48 | 15.0 | 1922 |  | 95 | 30.0 | 1999 | 4.0 |
|  |  |  |  |  |  |  |  |  |  |
| **Melanoma** |  | **394** | **100.0** | **3275** |  | **394** | **100.0** | **3421** | **4.5** |
| 3D-CRT | Standard | 99 | 25.0 | 3321 |  | 99 | 25.0 | 3663 | 10.3 |
| 3D-CRT | Hypofractionation | 99 | 25.0 | 2433 |  | 99 | 25.0 | 2657 | 9.2 |
| IMRT | Standard | 49 | 12.5 | 4385 |  | — |  |  | −100.0 |
| IMRT | Hypofractionation | 49 | 12.5 | 3180 |  |  |  |  | −100.0 |
| Rotational IMRT | Standard | 49 | 12.5 | 4122 |  | 99 | 25.0 | 4267 | 3.5 |
| Rotational IMRT | Hypofractionation | 49 | 12.5 | 3005 |  | 99 | 25.0 | 3098 | 3.1 |
| **Myeloma** |  | **56** | **100.0** | **3141** |  | **56** | **100.0** | **2670** | **−15.0** |
| 3D-CRT | Standard | - |  |  |  | 56 | 100.0 | 2670 |  |
| IMRT | Standard | 39 | 70.0 | 3194 |  | - |  |  | −100.0 |
| Rotational IMRT | Standard | 17 | 30.0 | 3019 |  | - |  |  | −100.0 |
| **Esophagus** |  | **190** | **100.0** | **4201** |  | **190** | **100.0** | **4223** | **0.5** |
| IMRT | Standard | 133 | 70.0 | 4274 |  | - |  |  | −100.0 |
| Rotational IMRT | Standard | 57 | 30.0 | 4029 |  | 190 | 100.0 | 4223 | 4.8 |
| **Pancreas** |  | **130** | **100.0** | **4645** |  | **130** | **100.0** | **3509** | **−24.4** |
| 3D-CRT | Standard | - |  |  |  | 23 | 17.5 | 4098 |  |
| IMRT | Standard | 65 | 50.0 | 4767 |  | 10 | 7.5 | 5055 | 6.0 |
| Rotational IMRT | Standard | 65 | 50.0 | 4522 |  | 33 | 25.0 | 4670 | 3.3 |
| Stereotactic tech. |  | - |  |  |  | 65 | 50.0 | 2491 |  |
| **Prostate** |  | **1,765** | **100.0** | **4215** |  | **1,765** | **100.0** | **4422** | **4.9** |
| IMRT | Standard | 124 | 7.0 | 4740 |  | 124 | 7.0 | 5115 | 7.9 |
| Rotational IMRT | Standard | 1,465 | 83.0 | 4317 |  | 1,465 | 83.0 | 4518 | 4.7 |
| Rotational IMRT | Hypofractionation | 177 | 10.0 | 3000 |  | 177 | 10.0 | 3132 | 4.4 |
| **Rectum** |  | **803** | **100.0** | **3075** |  | **803** | **100.0** | **3189** | **3.7** |
| IMRT | Standard | 169 | 21.0 | 3983 |  | 56 | 7.0 | 4290 | 7.7 |
| IMRT | Extreme hypofractionation | 72 | 9.0 | 1368 |  | 24 | 3.0 | 1453 | 6.2 |
| Rotational IMRT | Standard | 393 | 49.0 | 3750 |  | 506 | 63.0 | 3922 | 4.6 |
| Rotational IMRT | Extreme hypofractionation | 169 | 21.0 | 1324 |  | 217 | 27.0 | 1385 | 4.5 |
| **Soft Tissue** |  | **182** | **100.0** | **3311** |  | **182** | **100.0** | **3542** | **7.0** |
| 3D-CRT | Standard | 91 | 50.0 | 2913 |  | 91 | 50.0 | 3194 | 9.7 |
| IMRT | Standard | 46 | 25.0 | 3818 |  | 46 | 25.0 | 4061 | 6.4 |
| Rotational IMRT | Standard | 46 | 25.0 | 3600 |  | 46 | 25.0 | 3717 | 3.3 |
| **Stomach** |  | **75** | **100.0** | **3269** |  | **75** | **100.0** | **3693** | **13.0** |
| 3D-CRT | Standard | 38 | 50.0 | 2871 |  | 19 | 25.0 | 3215 | 12.0 |
| IMRT | Standard | 19 | 25.0 | 3777 |  | 19 | 25.0 | 4082 | 8.1 |
| Rotational IMRT | Standard | 19 | 25.0 | 3558 |  | 38 | 50.0 | 3738 | 5.1 |
| **Testis** |  | **13** | **100.0** | **2262** |  | **13** | **100.0** | **2490** | **10.1** |
| 3D-CRT | Standard | 7 | 50.0 | 1998 |  | 3 | 25.0 | 2165 | 8.4 |
| IMRT | Standard | 3 | 25.0 | 2593 |  | 3 | 25.0 | 2738 | 5.6 |
| Rotational IMRT | Standard | 3 | 25.0 | 2460 |  | 7 | 50.0 | 2528 | 2.8 |
| **Thyroid** |  | **13** | **100.0** | **4066** |  | **13** | **100.0** | **4173** | **2.6** |
| IMRT | Standard | 7 | 50.0 | 4187 |  | 1 | 10.0 | 4516 | 7.9 |
| Rotational IMRT | Standard | 7 | 50.0 | 3945 |  | 12 | 90.0 | 4135 | 4.8 |
| **Uterus** |  | **230** | **100.0** | **3435** |  | **230** | **100.0** | **3510** | **2.2** |
| IMRT | Standard | 115 | 50.0 | 3536 |  | 23 | 10.0 | 3795 | 7.3 |
| Rotational IMRT | Standard | 115 | 50.0 | 3335 |  | 207 | 90.0 | 3479 | 4.3 |
| **Vagina** |  | **77** | **100.0** | **3667** |  | **77** | **100.0** | **3772** | **2.9** |
| IMRT | Standard | 39 | 50.0 | 3777 |  | 8 | 10.0 | 4082 | 8.1 |
| Rotational IMRT | Standard | 39 | 50.0 | 3558 |  | 69 | 90.0 | 3738 | 5.1 |
| **Bone metastases** |  | **914** | **100.0** | **938** |  | **914** | **100.0** | **856** | **−8.8** |
| 3D-CRT | Extreme hypofractionation | 626 | 68.5 | 851 |  | 823 | 90.0 | 831 | −2.4 |
| IMRT | Extreme hypofractionation | 197 | 21.5 | 1119 |  | - |  |  | −100.0 |
| Rotational IMRT | Extreme hypofractionation | 85 | 9.2 | 965 |  | 53 | 5.8 | 1050 | 8.9 |
| Stereotactic techniques |  | 7 | 0.7 | 3393 |  | 39 | 4.3 | 1121 | −67.0 |
| **Brain metastases** |  | **573** | **100.0** | **1753** |  | **573** | **100.0** | **1942** | **10.8** |
| 3D-CRT | Extreme hypofractionation | 332 | 58.0 | 1595 |  | 258 | 45.0 | 1748 | 9.6 |
| Rotational IMRT | Extreme hypofractionation | 103 | 18.0 | 1597 |  | 132 | 23.0 | 1823 | 14.2 |
| Stereotactic techniques |  | 138 | 24.0 | 2250 |  | 183 | 32.0 | 2301 | 2.2 |
| **Other metastases** |  | **2167** | **100.0** | **1264** |  | **2167** | **100.0** | **1316** | **4.1** |
| 3D-CRT | Extreme hypofractionation | 1734 | 80.0 | 1208 |  | 1734 | 80.0 | 1268 | 5.0 |
| IMRT | Extreme hypofractionation | 217 | 10.0 | 1518 |  | 108 | 5.0 | 1571 | 3.5 |
| Rotational IMRT | Extreme hypofractionation | 217 | 10.0 | 1464 |  | 325 | 15.0 | 1485 | 1.5 |

3D-CRT: 3 Dimensional conformal RT, EBRT: external beam radiotherapy, IMRT: Intensity modulated RT, rotational IMRT: intensity-modulated rotational RT

**Table 9.** Mean cost of EBRT treatment, including additional tests and hospital overhead costs, 2018 (€)

| **Tumor site** | **Courses (n)** | **Average cost HERO model (€)** | **Additional tests (€)^1^** | **Fiducial markers (€)** | **Average cost (€)** | **Hospital overhead +15%** | **Overall average cost (€)** |
| --- | --- | --- | --- | --- | --- | --- | --- |
| Bladder | 124 | 4340 |  |  | 4340 | 651 | 4991 |
| Brain | 353 | 3740 | 211 |  | 3951 | 593 | 4544 |
| Breast | 3,543 | 3055 |  |  | 3055 | 458 | 3513 |
| Cervix | 162 | 3777 | 11 |  | 3788 | 568 | 4356 |
| Head and neck | 840 | 4351 | 55 |  | 4406 | 661 | 5067 |
| Leukemia | 31 | 1714 |  |  | 1714 | 257 | 1971 |
| Lung | 1,185 | 4296 | 69 | 15 | 4380 | 657 | 5037 |
| Lymphoma | 318 | 2279 |  |  | 2279 | 342 | 2621 |
| Melanoma | 394 | 3275 |  |  | 3275 | 491 | 3766 |
| Myeloma | 56 | 3141 |  |  | 3141 | 471 | 3612 |
| Esophagus | 190 | 4201 |  |  | 4201 | 630 | 4831 |
| Pancreas | 130 | 4645 |  |  | 4645 | 697 | 5342 |
| Prostate | 1,765 | 4215 |  | 15 | 4230 | 635 | 4865 |
| Rectum | 803 | 3075 |  |  | 3075 | 461 | 3536 |
| Soft tissue | 182 | 3311 |  |  | 3311 | 497 | 3808 |
| Stomach | 75 | 3269 |  |  | 3269 | 490 | 3759 |
| Testis | 13 | 2262 |  |  | 2262 | 339 | 2601 |
| Thyroid | 13 | 4066 |  |  | 4066 | 610 | 4676 |
| Uterus | 230 | 3435 | 11 |  | 3446 | 517 | 3962 |
| Vagina | 77 | 3667 | 11 |  | 3678 | 552 | 4229 |
| Bone metastases | 914 | 938 |  |  | 938 | 141 | 1079 |
| Brain metastases | 573 | 1753 |  |  | 1753 | 263 | 2016 |
| Other metastases | 2,167 | 1264 |  |  | 1264 | 190 | 1454 |
| **Total** | **14,138** | **2985** |  |  | **3003** | **450** | **3453** |

^1^ Additional tests for the planning:

- MRI: in 100% of the brain tumors with curative intent and in 5% of cervical, uterine and vaginal cancer cases; PET: in 10% of lung cancer cases and 8% of head and neck cases.
- Fiducial markers: in 100% of pancreatic tumors that must be treated with stereotactic body radiation therapy (SBRT), in 100% of lung tumors that must be treated with SBRT and in 5% of prostate tumors.
